# Oxidative balance score and mortality: mediating role of insulin resistance across age strata in the NHANES cohort

**DOI:** 10.3389/fnut.2025.1604696

**Published:** 2025-06-09

**Authors:** Lin Zhang, Heming Zhang, Hongxia Xiang, Jiachen Zhang, Wei Gong, Junjie Xv, Xue Yu

**Affiliations:** ^1^Department of Cardiology, Beijing Hospital, National Center of Gerontology, Institute of Geriatric Medicine, Chinese Academy of Medical Sciences, Beijing, China; ^2^College of Life Science, University of Chinese Academy of Sciences, Beijing, China; ^3^Department of Anesthesiology, The Second Affiliated Hospital of Air Force Medical University, Xi'an, China; ^4^Department of Anesthesiology, The 963 Hospital of the PLA Joint Logistics Support Force, Jiamusi, China; ^5^Peking University Health Science Center, Beijing, China; ^6^Health Service Department of the Guard Bureau of the General Office of the Central Committee of the Communist Party of China, Beijing, China

**Keywords:** oxidative balance score, insulin resistance, mortality, mediation analysis, NHANES

## Abstract

**Background:**

This study examined the association between oxidative balance score (OBS), a composite measure of oxidative/antioxidative factors, and mortality, while investigating insulin resistance (IR) indices as potential mediators using a nationally representative cohort.

**Methods:**

A cohort of 11,849 U. S. adults from NHANES (2007–2018) was analyzed. OBS integrated 16 dietary and 4 lifestyle components. Mortality risks (all-cause, cardiovascular, cancer) were assessed via weighted Cox models. Mediation analysis evaluated the indirect effects of five IR indices (TyG index, TG/HDL-C, HOMA-IR, eGDR, VAI) on OBS-mortality associations, with statistical validation of mediation effects. Analyses were stratified by age (<65 vs. ≥65 years) and adjusted for sociodemographic, behavioral, and clinical covariates.

**Results:**

Higher OBS reduced risks of all-cause (HR = 0.652, 95% CI: 0.525–0.81) and cardiovascular mortality (HR = 0.605, 95% CI: 0.408–0.898), but not cancer mortality. Innovatively, eGDR mediated 17% of OBS’s protective effect on all-cause mortality in adults <65 years, while TyG index and HOMA-IR showed weaker mediation. No IR mediation occurred in older adults (≥65 years).

**Conclusion:**

Higher OBS levels were inversely associated with all-cause and cardiovascular mortality, partially mediated by insulin resistance pathways. These findings highlight OBS as a potential prognostic indicator for mortality risk.

## 1 Introduction

Insulin resistance (IR), characterized by a reduced response to insulin in target tissues despite elevated insulin levels, plays a pivotal role in the development of several chronic conditions, including metabolic syndrome, atherosclerosis, and type 2 diabetes (1). The global prevalence of IR is rising at an alarming rate, driven by factors such as obesity, sedentary lifestyles, and unhealthy dietary habits (2). According to projections by the International Diabetes Federation (IDF), the number of individuals affected by diabetes is expected to reach 643 million by 2030 and 783 million by 2045 (3). Consequently, identifying effective interventions, particularly lifestyle modifications aimed at improving insulin sensitivity, is essential for enhancing global public health and reducing long-term healthcare expenditures.

Oxidative stress, defined as an imbalance between reactive oxygen species production and antioxidant defenses, contributes to cellular damage and the pathogenesis of chronic diseases (4), which is closely linked to IR and plays a key role in the progression of complications and poorer prognoses (5). Chronic, low-grade oxidative stress has been identified as a key factor in the development of IR and its associated microvascular complications (6). The oxidative balance score (OBS), a comprehensive measure of oxidative and antioxidant status derived from 16 nutrients and 4 lifestyle factors, has been shown to be inversely associated with the risk of diabetes and mortality in patients (7–9). Furthermore, oxidative stress can impair the expression and localization of GLUT-4, subsequently reducing insulin sensitivity in insulin-dependent cells such as adipocytes and myocytes (10). Therefore, individuals with higher OBS may experience declined IR, potentially alleviating mortality risk. However, the euglycemic hyperinsulinemic clamp technique is the gold standard for measuring insulin resistance. However, this approach’s high cost and invasive nature make it impractical for implementation in large clinical settings (11). And the predictive value of various IR indices based on commonly available lab tests and anthropometric measures varies in terms of prognosis. To our knowledge, there is no prior study to assess the effect of OBS on specific IR indicators including triglyceride-glucose (TyG) index, triglyceride/high-density lipoprotein cholesterol (TG/HDL-C), homeostatic model assessment of insulin resistance (HOMA-IR), estimated glucose disposal rate (eGDR), and visceral adiposity index (VAI). Moreover, the underlying mediation effects of these IR indicators on the association between OBS and mortality risk remain unexplored.

Therefore, this study aimed to investigate the associations of OBS with all-cause, cardiovascular, and cancer mortality, and further examine the potential mediating role of different IR indices in these relationships.

## 2 Methods

### 2.1 Data source and study population

**Figure 1:**
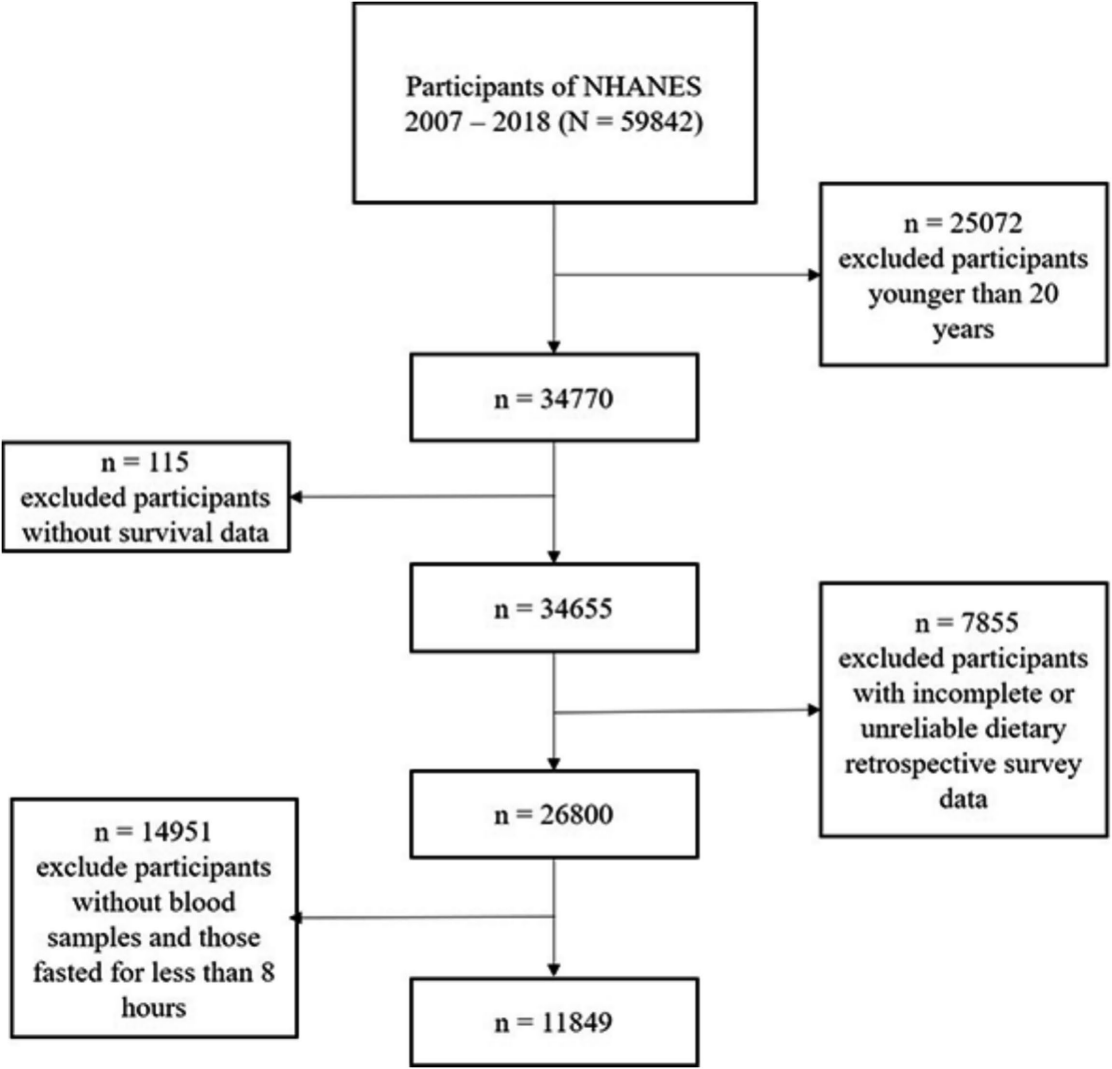
Enrollment flowchart. From the original 59,842 participants, 25,072 were excluded for age <20 years, 115 for missing survival data, 14,951 for incomplete blood samples or fasting time <8 h, and 7,855 for invalid dietary/OBS data, resulting in a final analytical cohort of 11,849 adults.

This cohort study utilized data from the National Health and Nutrition Examination Survey (NHANES), conducted by the National Center for Health Statistics (NCHS) under the Centers for Disease Control and Prevention (CDC). The analysis included participants from continuous NHANES cycles (2007–2018). Exclusion criteria were: (1) age <20 years; (2) missing mortality follow-up data; (3) incomplete blood sample collection or fasting time <8 h; and (4) invalid dietary survey data. The stepwise exclusion process is summarized in Figure 1. Ethical approval was granted by the NCHS Institutional Review Board, and all participants provided informed consent.

### 2.2 Oxidative balance score assessment

The Oxidative Balance Score (OBS) was used to assess oxidative stress, combining 16 dietary nutrients and 4 lifestyle factors (12). Of these, 15 antioxidants and 5 pro-oxidants were included in the dietary components. Nutrient intake was estimated from two 24-h dietary recall interviews. Lifestyle factors—physical activity (PA), body mass index (BMI), alcohol consumption, and smoking—were also considered, with smoking intensity assessed through cotinine levels, a biomarker for nicotine exposure. The full scoring methodology is provided in Supplementary Table S1 (12).

### 2.3 Insulin resistance indices assessment

The formulas for calculating diverse insulin resistance indices were as follows:

1. TyG index = Ln[fasting triglyceride (mg/dL) × fasting glucose (mg/dL)/2] (13);
2. TG/HDL-C = fasting triglyceride (mg/dL) / fasting high-density lipoprotein cholesterol (HDL-C; mg/dL) (14);
3. HOMA-IR = (fasting glucose [mmol/L] × fasting insulin [μU/mL]) / 22.5 (15);
4. eGDR = 21.158 – (0.09 × waist circumference [cm]) – (3.407 × hypertension [yes 1 or no 0]) – (0.551 × glycated hemoglobin A1c [HbA1c] [%]) (16);
5. VAI = male: (waist circumference [cm]/[39.68 + 1.88 × BMI]) × (fasting triglyceride [mmol/L]/1.03) × (1.31/fasting HDL-C [mmol/L]); female: (waist circumference [cm]/[36.58 + 1.89 × BMI]) × (fasting triglyceride [mmol/L]/0.81) × (1.52/fasting HDL-C [mmol/L]) (17).

### 2.4 Assessment of all-cause, cardiovascular, and cancer mortality

This study focused on all-cause, cardiovascular, and cancer mortality as primary outcomes (18, 19). Mortality data were obtained from the NHANES public-use linked mortality file, updated through December 31, 2019, and the National Death Index (NDI) using a probability matching algorithm. Causes of death were classified using the International Statistical Classification of Diseases, 10th Revision (ICD-10). Cardiovascular mortality was specifically identified by the NCHS as deaths due to heart disease, and cancer mortality was identified as deaths due to malignant neoplasms.

### 2.5 Covariates assessment

Several covariates were included in this analysis: sex, age (<65, ≥65), race/ethnicity (Non-Hispanic White, Non-Hispanic Black, other races), education level (high school or below, above high school), marital status (never married, married/living with partner, other), household income-to-poverty ratio (PIR: ≤1, 1 to ≤3.5, >3.5), household size (1 to 2, ≥3), sedentary duration (<5, 5 to <8, ≥8 h/day), sleep duration (<7, 7 to <8, ≥8 h/day), depression (PHQ-9: <10, ≥10), smoking status (never, former or current), and alcohol consumption (never, former or current).

### 2.6 Statistical analysis

This study employed NHANES analytic tutorials with appropriate weights to ensure that the estimates accurately reflect the US civilian noninstitutionalized population. All analyses accounted for sample weights, clustering, and stratification to estimate variance and maintain national representativeness. Missing data were addressed using multiple imputation via the R package “VIM.” Baseline characteristics were categorized according to OBS classification, with categorical variables reported as counts and percentages (%). Comparisons between categorical variables were conducted using the *χ*^2^ test. OBS and insulin resistance indices were grouped into two categories using the R package “survminer.” Kaplan–Meier curves were used to depict mortality rates across OBS and insulin resistance groups, with differences assessed by the log-rank test. The associations between OBS, insulin resistance, and mortality were evaluated using weighted univariate and multivariate Cox proportional hazards models, reporting hazard ratios (HRs) with 95% confidence intervals (CIs). Multiple linear regression models explored the relationships between OBS and insulin resistance indices, reporting Multiple linear regression models explored the relationships between OBS and insulin resistance indices, reporting beta coefficients (*β*) with 95% confidence intervals (CI). Model 1 was unadjusted, while Model 2 controlled for sex, age, and race. The fully adjusted model additionally controlled for education, marital status, PIR, household size, sedentary duration, sleep duration, depression, smoking, and alcohol use. Identical statistical methods were applied in subgroup analyses to explore potential differences across specific groups. Mediation analysis estimated indirect effects using the product of coefficients from (a) linear regression (OBS → insulin resistance indices) and (b) Cox models (insulin resistance → mortality). Bootstrap resampling derived 95% confidence intervals and *p*-values for mediation effects. Sensitivity analyses were performed by excluding participants who died within the first 24 months of follow-up to mitigate potential reverse causation bias. All statistical analyses were conducted using R (version 4.2.1), with a two-tailed p-value of <0.05 considered statistically significant.

## 3 Results

### 3.1 Baseline characteristics

Baseline characteristics stratified by OBS tertiles are shown in Table 1. A total of 11,849 participants were included in this analysis, with a weighted mean age of 47.8 years and 52.8 female. Participants were categorized into three OBS levels: Tertile 1 (<20), Tertile 2 (20 to <27), and Tertile 3 (≥27). Participants with higher OBS levels (Tertile 3) were generally younger, more likely to be Non-Hispanic White, more educated, married, and had a higher PIR (*p* < 0.001). Additionally, higher OBS levels were associated with a lower prevalence of smoking (*p* < 0.001). They were also linked to shorter sedentary time (*p* < 0.05), longer sleep duration (*p* < 0.001), and lower depression scores (*p* < 0.001).

**Table 1:** The demographic characteristics of the participants in the present study were stratified by OBS classification.

Characteristics	Total sample	OBS			*p*-value
		Tertile 1 (<20)	Tertile 2 (20 to <27)	Tertile 3 (≥27)	
Number of participants, n	11,849	4,032	3,788	4,029	
Gender, n (%)					0.233
Male	5,588 (47.16)	1895 (46.999)	1809 (47.756)	1884 (46.761)	
Female	6,261 (52.84)	2,137 (53.001)	1979 (52.244)	2,145 (53.239)	
Age (years), n (%)					<0.001
< 65	8,951 (75.542)	2,834 (70.288)	2,859 (75.475)	3,258 (80.864)	
≥ 65	2,898 (24.458)	1,198 (29.712)	929 (24.525)	771 (19.136)	
Race, n (%)					<0.001
Non-Hispanic White	5,149 (43.455)	1,637 (40.6)	1,671 (44.113)	1841 (45.694)	
Non-Hispanic Black	2,366 (19.968)	1,093 (27.108)	728 (19.219)	545 (13.527)	
Other Race	4,334 (36.577)	1,302 (32.292)	1,389 (36.668)	1,643 (40.779)	
Educational level, n (%)					<0.001
High school or below	5,390 (45.489)	2,298 (56.994)	1,676 (44.245)	1,416 (35.145)	
Above high school	6,459 (54.511)	1734 (43.006)	2,112 (55.755)	2,613 (64.855)	
Marital status, n (%)					<0.001
Never married	2062 (17.402)	752 (18.651)	626 (16.526)	684 (16.977)	
Married or living with partner	7,178 (60.579)	2,215 (54.936)	2,353 (62.117)	2,610 (64.78)	
Other	2,609 (22.019)	1,065 (26.414)	809 (21.357)	735 (18.243)	
PIR, n (%)					<0.001
<1	2,410 (20.339)	1,031 (25.57)	739 (19.509)	640 (15.885)	
1 to <3.5	5,851 (49.38)	2,193 (54.39)	1843 (48.654)	1815 (45.048)	
≥3.5	3,588 (30.281)	808 (20.04)	1,206 (31.837)	1,574 (39.067)	
Household size, n (%)					0.136
1–2	5,319 (44.89)	1843 (45.709)	1,677 (44.271)	1799 (44.651)	
≥3	6,530 (55.11)	2,189 (54.291)	2,111 (55.729)	2,230 (55.349)	
Sedentary (hours per day), n (%)					0.043
< 5	4,882 (41.202)	1,660 (41.171)	1,508 (39.81)	1714 (42.542)	
5 to <8	3,101 (26.171)	1,065 (26.414)	966 (25.502)	1,070 (26.557)	
≥ 8	3,866 (32.627)	1,307 (32.416)	1,314 (34.688)	1,245 (30.901)	
Sleep duration (hours per night), n (%)					<0.001
< 7	4,013 (33.868)	1,487 (36.88)	1,267 (33.448)	1,259 (31.248)	
7 to <8	5,970 (50.384)	1809 (44.866)	1931 (50.977)	2,230 (55.349)	
≥ 8	1866 (15.748)	736 (18.254)	590 (15.576)	540 (13.403)	
Depression (DPQ-9 score), n (%)					<0.001
< 10	10,836 (91.451)	3,553 (88.12)	3,488 (92.08)	3,795 (94.192)	
≥ 10	1,013 (8.549)	479 (11.88)	300 (7.92)	234 (5.808)	
Smoking					<0.001
Never	6,659 (56.199)	1946 (48.264)	2,148 (56.705)	2,565 (63.663)	
Former or current	5,190 (43.801)	2086 (51.736)	1,640 (43.295)	1,464 (36.337)	
Drinking					0.204
Never	1,536 (12.963)	542 (13.442)	442 (11.668)	552 (13.701)	
Former or current	10,313 (87.037)	3,490 (86.558)	3,346 (88.332)	3,477 (86.299)	

OBS, oxidative balance score; PIR, poverty-income ratio.

### 3.2 Associations of OBS with mortality

The associations between OBS and various mortality outcomes, including all-cause, cardiovascular, and cancer mortality, are summarized in Table 2. For all-cause mortality, a higher OBS was associated with a significantly reduced risk of death across all models. Participants in the highest OBS tertile (Tertile 3) demonstrated a markedly lower mortality risk (HR: 0.652, 95% CI: 0.525–0.81, *p* < 0.001) compared to those in the lowest tertile (Tertile 1). These findings were consistent with the Kaplan–Meier survival curves in Supplementary Figure S2A, where participants with higher OBS levels exhibited significantly improved survival rates (*p* < 0.001).

**Table 2:** The associations of OBS with all-cause mortality and cardiovascular mortality in the US participants.

Outcomes	Model 1		Model 2		Model 3	
	HR (95% CI)	*p*-value	HR (95% CI)	*p*-value	HR (95% CI)	*p*-value
All-cause mortality						
OBS	0.945 (0.934 to 0.956)	< 0.001	0.952 (0.941 to 0.963)	< 0.001	0.973 (0.961 to 0.986)	< 0.001
OBS category						
Tertile 1	Reference		Reference		Reference	
Tertile 2	0.63 (0.511 to 0.776)	< 0.001	0.677 (0.555 to 0.826)	< 0.001	0.82 (0.662 to 1.015)	0.069
Tertile 3	0.385 (0.315 to 0.47)	< 0.001	0.463 (0.377 to 0.57)	< 0.001	0.652 (0.525 to 0.81)	< 0.001
Cardiovascular mortality						
OBS	0.944 (0.927 to 0.961)	< 0.001	0.953 (0.935 to 0.972)	< 0.001	0.974 (0.954 to 0.995)	0.014
OBS category						
Tertile 1	Reference		Reference		Reference	
Tertile 2	0.719 (0.49 to 1.053)	0.09	0.787 (0.548 to 1.13)	0.194	0.96 (0.648 to 1.422)	0.837
Tertile 3	0.339 (0.231 to 0.496)	< 0.001	0.427 (0.291 to 0.625)	< 0.001	0.605 (0.408 to 0.898)	0.013
Cancer mortality						
OBS	0.96 (0.942 to 0.979)	< 0.001	0.968 (0.949 to 0.987)	0.001	0.986 (0.966 to 1.006)	0.158
OBS category						
Tertile 1	Reference		Reference		Reference	
Tertile 2	0.65 (0.452 to 0.934)	0.02	0.695 (0.477 to 1.011)	0.057	0.806 (0.555 to 1.171)	0.258
Tertile 3	0.461 (0.331 to 0.643)	< 0.001	0.551 (0.384 to 0.791)	0.001	0.724 (0.499 to 1.049)	0.088

OBS, oxidative balance score; HR, hazard ratio; CI, confidence interval. Model 1: not adjusted for covariates. Model 2: adjusted for sex, age, and race. Model 3: adjusted for sex, age, race, education, marital status, PIR, household size, sedentary duration, sleep duration, depression, smoking, and drinking.

For cardiovascular mortality, a higher OBS was similarly linked to reduced risk in the fully adjusted model (*p* = 0.014). Participants in Tertile 3 showed a significantly lower risk compared to those in Tertile 1 (HR: 0.605, 95% CI: 0.408–0.898, *p* = 0.013). This association is further supported by the Kaplan–Meier curves in Supplementary Figure S3A, where higher OBS levels correlated with improved cardiovascular survival rates (*p* < 0.001). For cancer mortality, no significant association was observed after full adjustment. While the survival curves in Supplementary Figure S4A illustrate the significant improvement between the higher and lower OBS groups (*p* < 0.001).

### 3.3 Subgroup analysis of OBS and mortality

Subgroup analyses revealed that the protective associations between OBS and mortality outcomes varied across demographic and lifestyle factors. For all-cause mortality, higher OBS levels were consistently associated with lower risk across most subgroups. The effect was stronger in participants aged <65, males, Non-Hispanic White, and those with higher education or socioeconomic status. Lifestyle factors, such as nonsmoking and shorter sedentary time, also amplified the protective effect of OBS (Supplementary Table S2). Similar patterns were observed for cardiovascular mortality. The protective association was more pronounced in males, Non-Hispanic White, and participants aged ≥65 years. In contrast, the association was weaker and nonsignificant in other racial groups and some lifestyle subgroups, such as those with longer sedentary time (Supplementary Table S3). For cancer mortality, the associations were generally weaker and mostly nonsignificant across subgroups (Supplementary Table S4). While some trends suggested a protective effect of higher OBS in specific subgroups (e.g., Non-Hispanic White and nonsmokers), the results lacked statistical significance in fully adjusted models.

### 3.4 Associations of insulin resistance indices with OBS and mortality

The associations between OBS and insulin resistance indices are shown in Table 3. Participants in the highest OBS tertile (Tertile 3) exhibited significantly lower levels of insulin resistance, as reflected by reductions in TyG index (*β* = −0.121, 95% CI: −0.163 to-0.079, *p* < 0.001), TG/HDL-C ratio (*β* = −0.304, 95% CI: −0.492 to-0.116, *p* = 0.002), HOMA-IR (*β* = −0.836, 95% CI: −1.13 to −0.541, *p* < 0.001), and VAI (*β* = −0.113, 95% CI: −0.176 to −0.049, *p* < 0.001). eGDR was significantly higher in Tertile 3 (*β* = 0.562, 95% CI: 0.398 to 0.726, *p* < 0.001). Stratified analyses indicated that these associations were more pronounced in participants aged <65 years, particularly for HOMA-IR (*β* = −0.914, 95% CI: −1.289 to −0.538, *p* < 0.001). In participants aged ≥65 years, the associations between OBS and HOMA-IR, TG/HDL-C ratio, and VAI were weaker and not statistically significant (*p* > 0.05), suggesting that age may modulate the relationship between OBS and insulin resistance indices. Supplementary Tables S5–S7 provide additional analyses using less-adjusted models, which were broadly consistent with the fully adjusted models.

**Table 3:** The associations between OBS and insulin resistance indices.

IR indices	Total sample		< 65 years		≥ 65 years	
	β (95% CI)	*p*-value	β (95% CI)	*p*-value	β (95% CI)	*p*-value
TyG index						
OBS tertile 1	Reference		Reference		Reference	
OBS tertile 2	-0.04 (−0.08 to-0.001)	0.048	−0.041 (−0.09 to 0.007)	0.095	−0.039 (−0.098 to 0.019)	0.186
OBS tertile 3	−0.121 (−0.163 to-0.079)	< 0.001	−0.122 (−0.175 to-0.068)	< 0.001	−0.127 (−0.215 to-0.038)	0.005
TG/HDL-C						
OBS tertile 1	Reference		Reference		Reference	
OBS tertile 2	−0.059 (−0.247 to 0.129)	0.534	−0.041 (−0.264 to 0.182)	0.712	−0.179 (−0.431 to 0.072)	0.16
OBS tertile 3	−0.304 (−0.492 to-0.116)	0.002	−0.325 (−0.557 to-0.093)	0.007	−0.27 (−0.581 to 0.041)	0.088
HOMA-IR						
OBS tertile 1	Reference		Reference		Reference	
OBS tertile 2	−0.307 (−0.646 to 0.033)	0.076	−0.337 (−0.788 to 0.113)	0.14	−0.259 (−1.11 to 0.593)	0.547
OBS tertile 3	−0.836 (−1.13 to-0.541)	< 0.001	−0.914 (−1.289 to-0.538)	< 0.001	−0.542 (−1.285 to 0.201)	0.151
eGDR						
OBS tertile 1	Reference		Reference		Reference	
OBS tertile 2	0.051 (−0.116 to 0.218)	0.543	0.035 (−0.156 to 0.226)	0.716	0.059 (−0.196 to 0.314)	0.644
OBS tertile 3	0.562 (0.398 to 0.726)	< 0.001	0.533 (0.343 to 0.723)	< 0.001	0.639 (0.352 to 0.927)	< 0.001
VAI						
OBS tertile 1	Reference		Reference		Reference	
OBS tertile 2	−0.023 (−0.088 to 0.042)	0.485	−0.017 (−0.094 to 0.061)	0.671	−0.062 (−0.157 to 0.033)	0.195
OBS tertile 3	−0.113 (−0.176 to-0.049)	0.001	−0.117 (−0.195 to-0.038)	0.004	−0.109 (−0.221 to 0.003)	0.057

TyG index, triglyceride-glucose index; TG/HDL-C, triglyceride/high-density lipoprotein cholesterol ratio; HOMA-IR, homeostatic model assessment of insulin resistance; eGDR, estimated glucose disposal rate; VAI, visceral adiposity index; CI, confidence interval.

Supplementary Figures S1B–F illustrates the cutpoints for the insulin resistance indices used in the stratified analyses. The associations between insulin resistance indices and mortality outcomes are summarized in Table 4. Across the total sample, higher HOMA-IR was significantly associated with an increased risk of all-cause mortality (HR = 1.012, 95% CI: 1.005–1.019, *p* = 0.001) and cardiovascular mortality (HR = 1.014, 95% CI: 1.001–1.028, *p* = 0.035). Similarly, a higher TyG index showed a suggestive but nonsignificant trend for increased risk of all-cause mortality (HR = 1.148, 95% CI: 0.994–1.324, *p* = 0.06) and cardiovascular mortality (HR = 1.177, 95% CI: 0.958–1.43, *p* = 0.124). In contrast, eGDR was inversely associated with both all-cause mortality (HR = 0.844, 95% CI: 0.728–0.979, *p* = 0.026) and cardiovascular mortality (HR = 0.844, 95% CI: 0.728–0.979, *p* = 0.026). Stratified analyses showed that these associations were more pronounced in participants aged <65 years. In this group, higher TyG index was significantly associated with increased risk of all-cause mortality (HR = 1.536, 95% CI: 1.179–2.001, *p* < 0.001), while higher eGDR was associated with reduced risk (HR = 0.778, 95% CI: 0.584–0.973, *p* = 0.029). Among participants aged ≥65 years, the associations were generally weaker and not statistically significant (*p* > 0.05). Less-adjusted models were shown in Supplementary Tables S8–S10.

**Table 4:** The associations of insulin resistance indices with mortality.

IR indices	Total sample		< 65 years		≥ 65 years	
	HR (95% CI)	*p*-value	HR (95% CI)	*p*-value	HR (95% CI)	*p*-value
All-cause mortality						
TyG index	1.148 (0.994 to 1.324)	0.06	1.536 (1.179 to 2.001)	0.001	0.959 (0.794 to 1.156)	0.658
TG/HDL-C	1.001 (0.983 to 1.019)	0.934	1.005 (0.989 to 1.021)	0.51	0.999 (0.942 to 1.06)	0.983
HOMA-IR	1.012 (1.005 to 1.019)	0.001	1.019 (1.011 to 1.027)	< 0.001	1 (0.983 to 1.018)	0.978
eGDR	0.909 (0.878 to 0.94)	< 0.001	0.827 (0.78 to 0.877)	< 0.001	0.964 (0.922 to 1.007)	0.099
VAI	1.015 (0.974 to 1.057)	0.49	1.009 (0.968 to 1.052)	0.681	0.961 (0.796 to 1.161)	0.68
Cardiovascular mortality						
TyG index	1.17 (0.958 to 1.43)	0.124	1.872 (1.199 to 2.922)	0.006	0.884 (0.695 to 1.124)	0.314
TG/HDL-C	0.978 (0.93 to 1.028)	0.375	0.994 (0.956 to 1.033)	0.746	0.973 (0.869 to 1.089)	0.63
HOMA-IR	1.014 (1.001 to 1.028)	0.031	1.022 (1.011 to 1.034)	< 0.001	1 (0.968 to 1.033)	0.997
eGDR	0.844 (0.788 to 0.905)	< 0.001	0.7 (0.602 to 0.813)	< 0.001	0.916 (0.85 to 0.987)	0.021
VAI	0.933 (0.815 to 1.069)	0.318	0.968 (0.824 to 1.137)	0.694	0.777 (0.568 to 1.064)	0.116
Cancer mortality						
TyG index	1.021 (0.81 to 1.287)	0.859	1.173 (0.827 to 1.665)	0.371	0.974 (0.716 to 1.327)	0.868
TG/HDL-C	0.983 (0.94 to 1.028)	0.456	0.987 (0.944 to 1.032)	0.564	1.002 (0.924 to 1.086)	0.969
HOMA-IR	1.002 (0.985 to 1.02)	0.828	1 (0.962 to 1.039)	0.984	1.003 (0.984 to 1.024)	0.741
eGDR	0.985 (0.927 to 1.047)	0.632	0.955 (0.885 to 1.03)	0.234	0.996 (0.921 to 1.077)	0.925
VAI	0.974 (0.861 to 1.102)	0.677	0.966 (0.838 to 1.113)	0.631	0.978 (0.763 to 1.253)	0.859

TyG index, triglyceride-glucose index; TG/HDL-C, triglyceride/high-density lipoprotein cholesterol ratio; HOMA-IR, homeostatic model assessment of insulin resistance; eGDR, estimated glucose disposal rate; VAI, visceral adiposity index; CI, confidence interval.

### 3.5 Mediating role of insulin resistance indices

**Figure 2:**
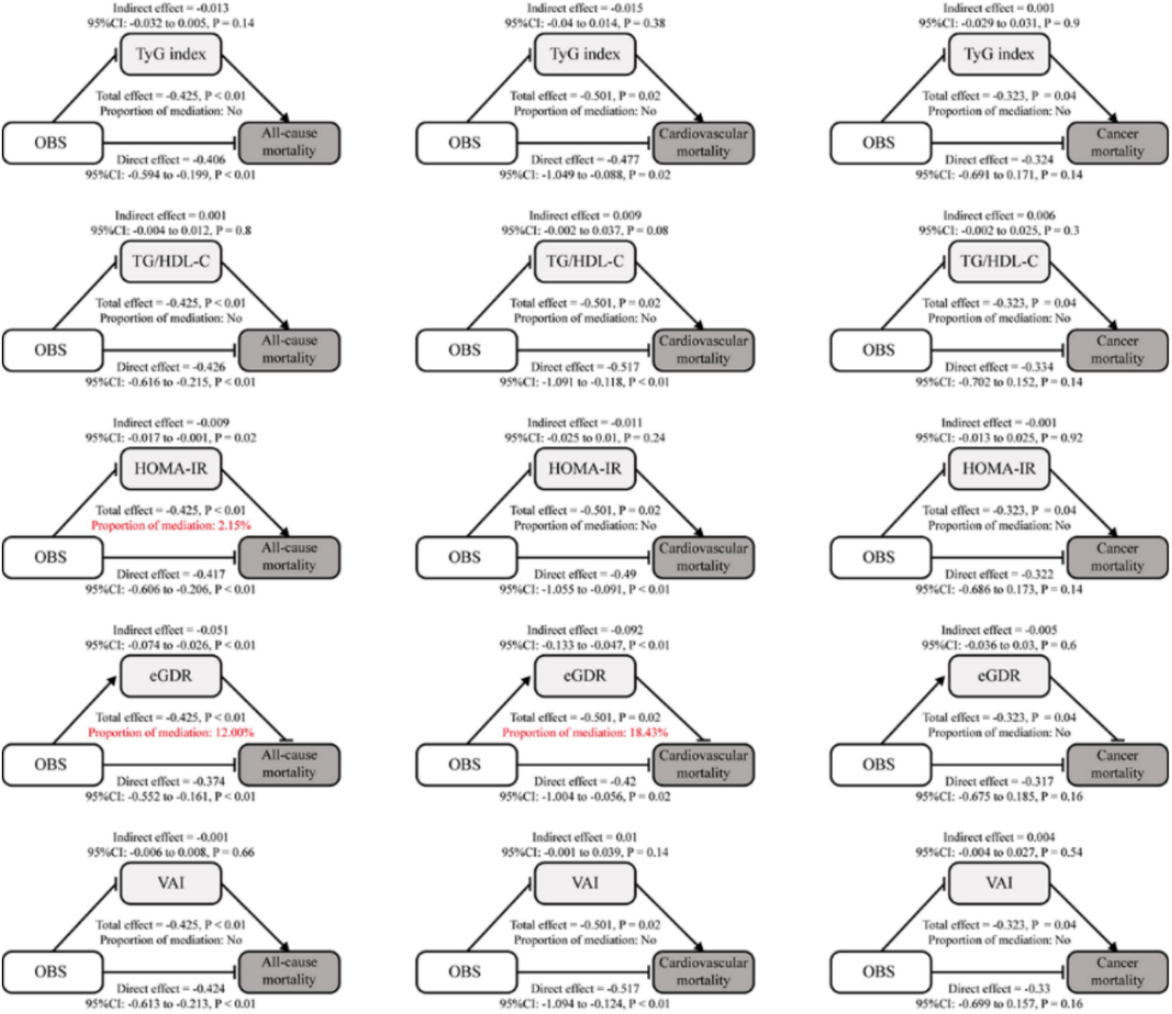
Analysis of the mediation by insulin resistance indices of the associations of OBS with mortality. Mediation analysis of the associations between OBS and mortality outcomes (all-cause, cardiovascular, and cancer) through insulin resistance indices. Significant mediation effects were highlighted in red for TG/HDL-C and eGDR in all-cause mortality and eGDR in cardiovascular mortality.

Mediation analysis revealed the role of insulin resistance indices in the relationship between OBS and mortality outcomes. Figure 2 presents the mediation effects of key insulin resistance indices in the overall population, showing that HOMA-IR and eGDR mediated 2.15 and 12.00% of the total effect of OBS on all-cause mortality, respectively, while only eGDR showed a significant mediating effect (18.43%) on cardiovascular mortality. These effects were further assessed in younger (<65 years; Supplementary Figure S5) and older (≥65 years, Supplementary Figure S6) populations. In the <65 years group, eGDR mediated 16.16% of the total effect on all-cause mortality (indirect effect = −0.057, *p* < 0.01) and 19.33% on cardiovascular mortality (indirect effect = −0.882, *p* < 0.05). In contrast, in the ≥65 years group, the mediation effects of eGDR were nonsignificant for both all-cause and cardiovascular mortality, suggesting a more prominent role in younger individuals.

HOMA-IR and TyG index also showed modest mediation effects in the <65 years group, mediating 2.68% (indirect effect = −0.571, *p* < 0.01) and 8.32% (indirect effect = −0.571, *p* < 0.01) of the total effect on all-cause mortality, respectively. However, their effects on cardiovascular and cancer mortality were nonsignificant (*p* > 0.05). In the ≥65 years group, neither the HOMA-IR nor the TyG index showed significant mediation effects for any outcomes. Other indices, including TG/HDL-C ratio and VAI, exhibited negligible mediating roles across all outcomes and age groups, with nonsignificant indirect effects.

### 3.6 Sensitivity analyses

Sensitivity analyses were performed on the population of participants who survived the first 24 months of follow-up, excluding those who died within this period to mitigate potential reverse causation bias. These analyses confirmed the robustness of the mediating effects of insulin resistance indices in the associations between OBS and mortality outcomes. eGDR consistently demonstrated significant mediation, particularly for all-cause and cardiovascular mortality, with proportions mediated reaching 12.99 and 17.03% for all-cause mortality and 15.17 and 14.70% for cardiovascular mortality (Supplementary Figures S7, S8). While, indices such as HOMA-IR, TyG index, TG/HDL-C ratio, and VAI showed negligible or nonsignificant mediation effects for all-cause and cardiovascular mortality, as indicated in Supplementary Figure S9. Moreover, no indices displayed substantial mediation for cancer mortality across all models (Supplementary Figures S7–S9). These findings underscore the stability of eGDR as a key mediator across different models, while other indices contributed minimally or inconsistently to the mediation effects.

## 4 Discussion

Our study demonstrated a positive correlation of OBS with both all-cause and cardiovascular mortality, findings which persisted even after full model adjustments. In contrast, no significant link was found between OBS and cancer mortality. Analysis of the relationships between IR indicators and mortality showed that, for participants under 65, mediation analysis identified the TyG index, HOMA-IR, and eGDR as important mediators in the association between OBS and all-cause mortality. Additionally, the TyG index and eGDR were implicated in mediating the link between OBS and cardiovascular mortality. Conversely, no significant mediating effect of IR was observed in participants aged 65 and older. These findings imply that IR could be a potential mechanism underlying these relationships, and that OBS monitoring might serve as a practical tool for epidemiological research into adverse health outcomes.

Previous research has highlighted the potential advantages of OBS in enhancing clinical outcomes. For instance, Talavera-Rodriguez et al. reported that higher OBS levels were linked to lower mortality from all causes, cardiovascular events, and cancer (20). In contrast, our findings did not reveal a significant association between OBS and cancer mortality, differing partially from Talavera-Rodriguez’s results. This discrepancy might stem from the different selection of age ranges among the study participants, as the average age of the dynamic cohort of Spanish university graduates in Talavera-Rodriguez’s study was approximately 20 years younger than the subjects in this study. Additionally, some previous studies have explored the relationship between OBS and clinical outcomes in patients with various diseases. Xu et al. found that individuals with diabetes or prediabetes with higher OBS levels had reduced risks of all-cause and cardiovascular mortality (7). Xu et al. also observed that OBS was negatively associated with all-cause mortality in participants with metabolic syndrome (21). Moreover, evidence suggested that OBS was linked to a reduced risk of all-cause mortality in *H. pylori* uninfected patients but not in those without *H. pylori* uninfected (22). Our study provides additional evidence supporting the role of OBS in all-cause and cardiovascular mortality risk in general individuals and further indicates that this link could be influenced by many factors such as race, marital status, and PIR. Notably, the effect of OBS on cardiovascular mortality risk was only observed in male participants or those aged at least 65 years in this study.

To the best of our knowledge, this is the first study to explore the impact of OBS on IR indicators using a nationally representative dataset. Previous research has only noted an inverse relationship between OBS and metabolic syndrome, a comprehensive measure encompassing waist circumference, fasting plasma glucose, triglycerides, high-density lipoprotein, and blood pressure (23). In our analysis, we examined the associations between OBS and various IR indicators, including the TyG index, TG/HDL-C, HOMA-IR, eGDR, and VAI. The results demonstrated that higher OBS was linked to improvements in IR status. Notably, the associations for TG/HDL-C, HOMA-IR, and VAI were significant only in participants under 65 years of age. Additionally, we investigated the mediating role of these IR indicators in the relationship between OBS and mortality risk. The mediation analysis provided insights into potential pathways influencing mortality, offering more comprehensive survival guidance for the population. Specifically, our findings indicated that increased OBS could significantly decrease the risks of all-cause and cardiovascular mortality, mediated by TyG index, HOMA-IR, and eGDR. Of these, HOMA-IR showed a relatively minor mediating role in the link between OBS and cardiovascular mortality, which could be influenced by short-term mortality risk. Furthermore, our results emphasized the critical role of age, revealing that the mediating effects of IR indicators on mortality outcomes were significant only in participants younger than 65.

Several potential biological pathways might explain our findings. In the aspect of lifestyle factors, evidence suggested that physical activity could increase the biogenesis of muscle mitochondrial, GLUT4 protein content and glucose uptake and promote the repartition of IMCL, which could further enhance insulin sensitivity (24). Smoking has been shown to promote inflammation by activating inflammatory cytokines like TNF-*α* and IL-6, which interfere with the normal function of insulin signaling (25). Alcohol consumption induces mitochondrial dysfunction, reducing ATP production, impairing insulin responsiveness, and promoting the accumulation of toxic metabolites that exacerbate insulin resistance (26). Moreover, many dietary oxidants and antioxidants were confirmed to be related to IR. For instance, an oversupply of fatty acid can translocate to the mitochondrial matrix via a flip-flop mechanism, which bypasses both acyl-CoA synthase and carnitine palmitoyl acyl transferase 1 and then results in IR (27). Omega-3 polyunsaturated fatty acids can prevent or reverse the impairments in skeletal muscle mitochondrial function by increasing fatty acid oxidation (28). Additionally, caloric restriction can increase mitochondrial biogenesis and efficiency while reducing mitochondrial production of reactive oxygen species (ROS) (27).

Our study presents several notable strengths. Firstly, it is the first research to investigate the relationship between OBS and IR indicators, as well as to examine the mediating role of these IR indicators in the link between OBS and mortality outcomes. Secondly, by employing sample weights, clustering, and stratification, we ensured accurate variance estimation and national representativeness of the U. S. population, enhancing the applicability of our results. Additionally, sensitivity analyses were performed to confirm the robustness of our primary findings.

Nonetheless, certain limitations should be acknowledged and addressed in future research. Primarily, the data on OBS components, such as daily diet and physical activity, were self-reported, which may have introduced recall bias. Specifically, the 24-h dietary recall methodology—relying on participants’ memory to report food intake—is prone to inaccuracies from recall bias (e.g., underreporting of energy-dense foods and overreporting of socially desirable items) and day-to-day intake variability. These measurement errors could attenuate diet-outcome associations. Moreover, the study assessed OBS and IR indicators only at baseline, without tracking changes over the follow-up period. Lastly, since the NHANES dataset is specific to the US population, further multicenter research is necessary to confirm the external validity of our results, thereby broadening their generalizability and clinical relevance.

## 5 Conclusion

The present study provided evidence for the relationship between OBS and IR indicators, while also highlighting the significant mediating role of IR indicators in the association between OBS and all-cause and cardiovascular mortality outcomes in participants younger than 65 years. These insights add to the growing evidence supporting the clinical utility of OBS in prognosis prediction and contribute valuable perspectives for the development of intervention strategies in diverse populations.

## Data Availability

Publicly available datasets were analyzed in this study. This data can be found at: https://www.cdc.gov/nchs/nhanes/.

## Supplementary material

The Supplementary material for this article can be found online at:

https://www.frontiersin.org/articles/10.3389/fnut.2025.1604696/full#supplementary-material
