# Supplementary material for: Oxidative balance score and mortality: mediating role of insulin resistance across age strata in the NHANES cohort
Source: Front Nutr. 2025 Jun 9;12:1604696. doi: 10.3389/fnut.2025.1604696 (PMC12184654; doi:10.3389/fnut.2025.1604696)
Supplement: Supplementary file 1 [file Data_Sheet_1.zip › Supplementary materials/Figure S1-S9.docx]

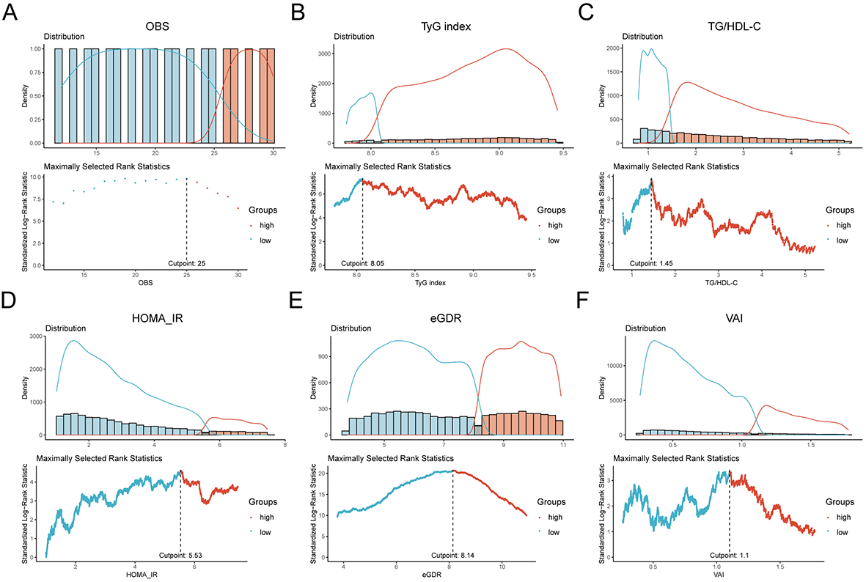


Figure S1. The cutpoints for the insulin resistance indices

Cutpoints for insulin resistance indices (A: OBS, B: TyG Index, C: TG/HDL-C, D: HOMA-IR, E: eGDR, F: VAI) determined by maximally selected rank statistics. Each panel shows the distribution of the respective index and the identified cutpoint dividing high and low groups for further analysis.


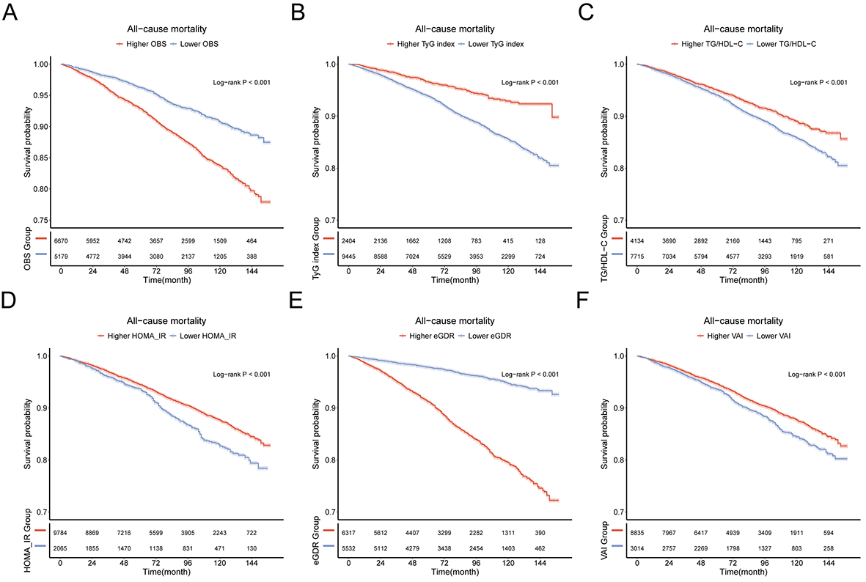


Figure S2. Survival Analysis of OBS and Insulin Resistance Indices on All-Cause Mortality

Kaplan-Meier survival curves illustrating the impact of OBS (A) and insulin resistance indices (B: TyG Index, C: TG/HDL-C, D: HOMA-IR, E: eGDR, F: VAI) on all-cause mortality. Higher levels of OBS and insulin resistance indices, except for eGDR (where lower levels are associated), are significantly associated with increased mortality (log-rank P < 0.001).


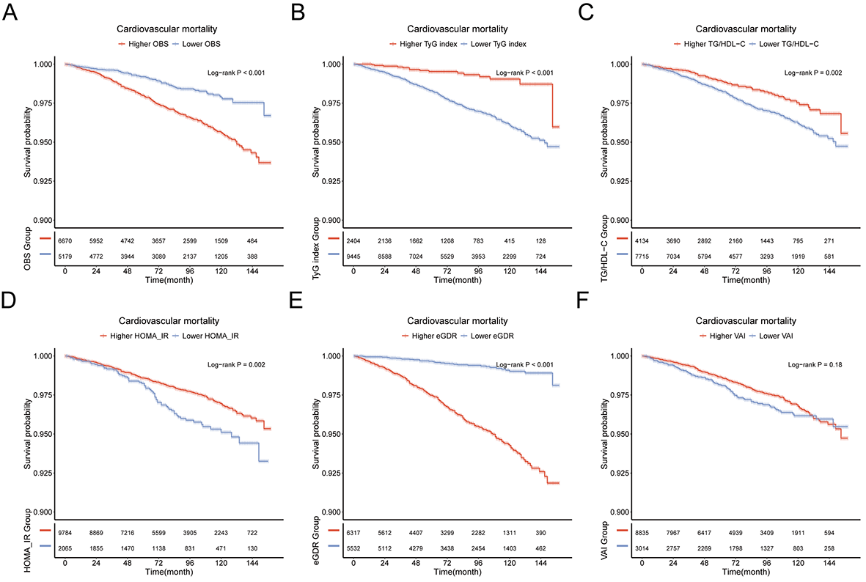


Figure S3. Survival Analysis of OBS and Insulin Resistance Indices on Cardiovascular Mortality

Kaplan-Meier survival curves showing the association of OBS (A) and insulin resistance indices (B: TyG Index, C: TG/HDL-C, D: HOMA-IR, E: eGDR, F: VAI) with cardiovascular mortality. Higher levels of OBS and insulin resistance indices, except for eGDR (where lower levels are associated), are significantly correlated with increased cardiovascular mortality (log-rank P < 0.001), except for VAI (F) which shows no significant association (log-rank P= 0.18).


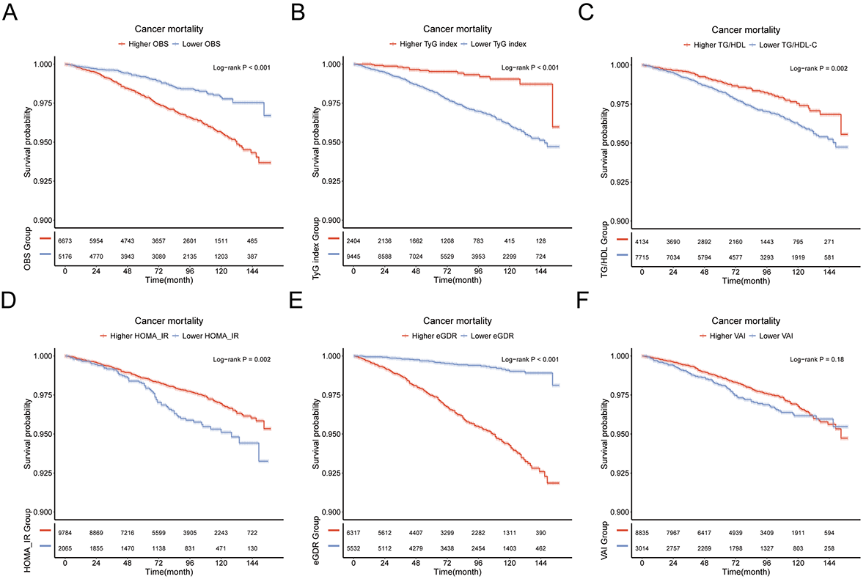


Figure S4. Survival Analysis of OBS and Insulin Resistance Indices on Cancer Mortality

Kaplan-Meier survival curves assessing the relationship between OBS (A) and insulin resistance indices (B: TyG Index, C: TG/HDL-C, D: HOMA-IR, E: eGDR, F: VAI) with cancer mortality. Higher OBS and insulin resistance indices, except for eGDR (where lower levels are associated), are significantly linked to increased cancer mortality (log-rank P < 0.001), while VAI (F) shows no significant association (log-rank P = 0.51).


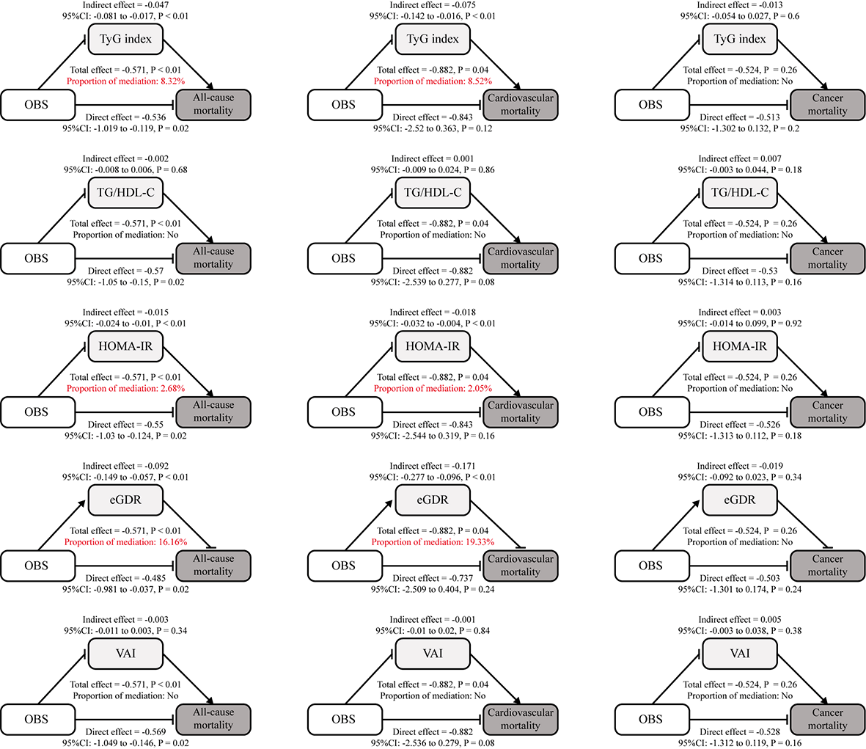


Figure S5: Mediation Analysis of Insulin Resistance Indices in the Association Between OBS and Mortality Among Participants Younger Than 65 Years

Mediation analysis of the associations between OBS and mortality outcomes (all-cause, cardiovascular, and cancer) in participants younger than 65 through insulin resistance indices. Significant mediation effects, highlighted in red, were observed for TyG index, TG/HDL-C, HOMA-IR, and eGDR in all-cause mortality and cardiovascular mortality (all P < 0.05).


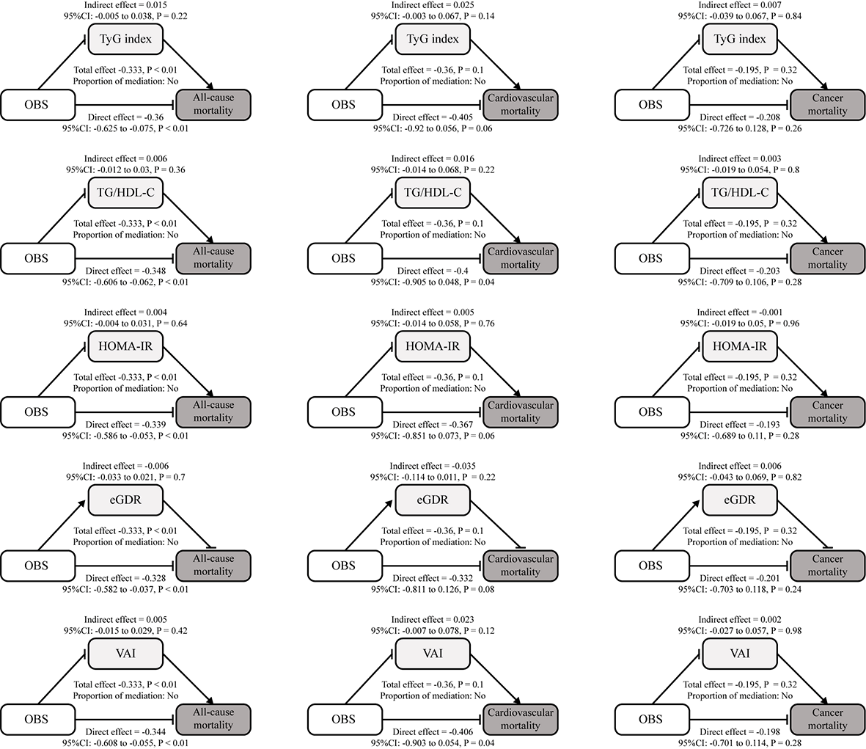


Figure S6: Analysis of the mediation by insulin resistance indices of the associations of OBS with mortality in participants at least 65 years old.

Mediation analysis of the associations between OBS and mortality outcomes (all-cause, cardiovascular, and cancer) in participants aged 65 years or older through insulin resistance indices. No significant mediation effects were observed for any insulin resistance indices in this age group (all P ≥ 0.05).


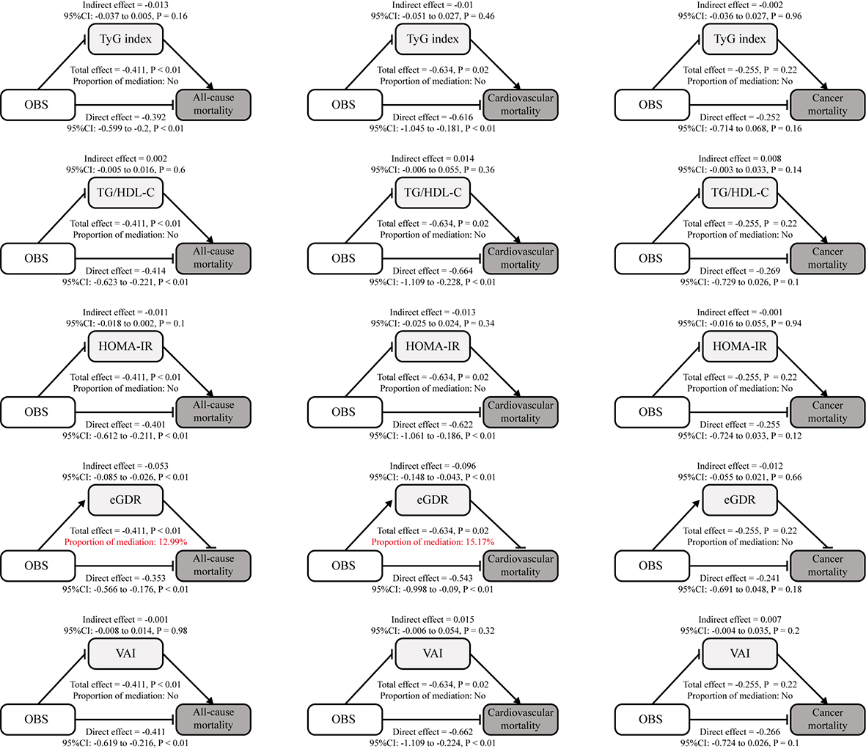


Figure S7: Sensitivity analysis of the mediation by insulin resistance indices of the associations of OBS with mortality.

Sensitivity analysis of the mediation by insulin resistance indices in the associations between OBS and mortality outcomes (all-cause, cardiovascular, and cancer). Significant mediation effects, highlighted in red, were observed for eGDR in both all-cause and cardiovascular mortality (P < 0.05). No significant mediation effects were observed for other indices.


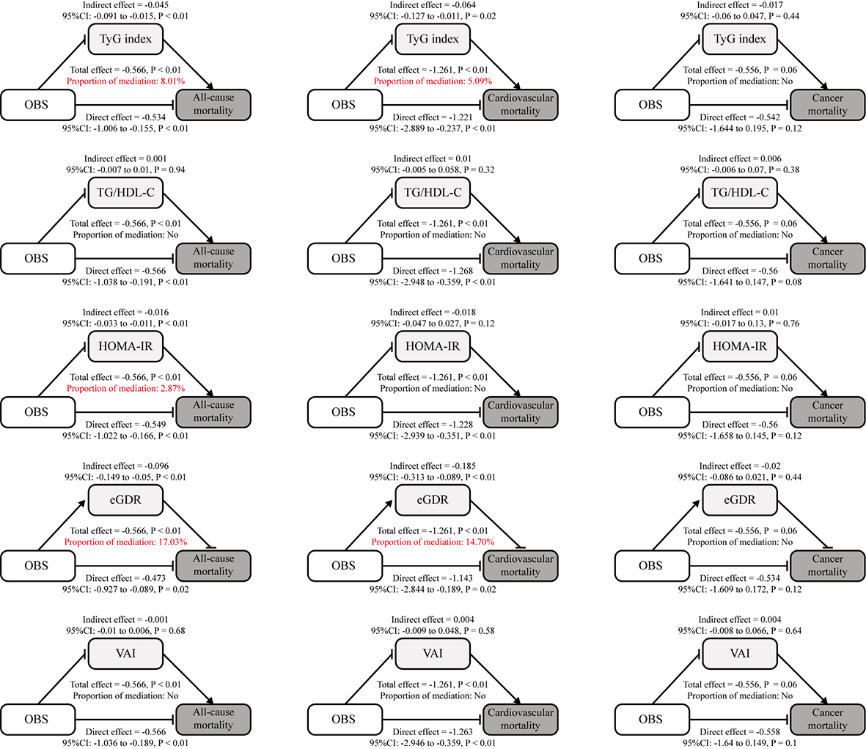


Figure S8: Sensitivity analysis of the mediation by insulin resistance indices of the associations of OBS with mortality in participants younger than 65 years

Sensitivity analysis of the mediation by insulin resistance indices in the associations between OBS and mortality outcomes (all-cause, cardiovascular, and cancer) in participants younger than 65. Significant mediation effects were highlighted in red for TyG index, HOMA-IR, and eGDR in all-cause mortality, and TyG index and eGDR in cardiovascular mortality (P < 0.05).


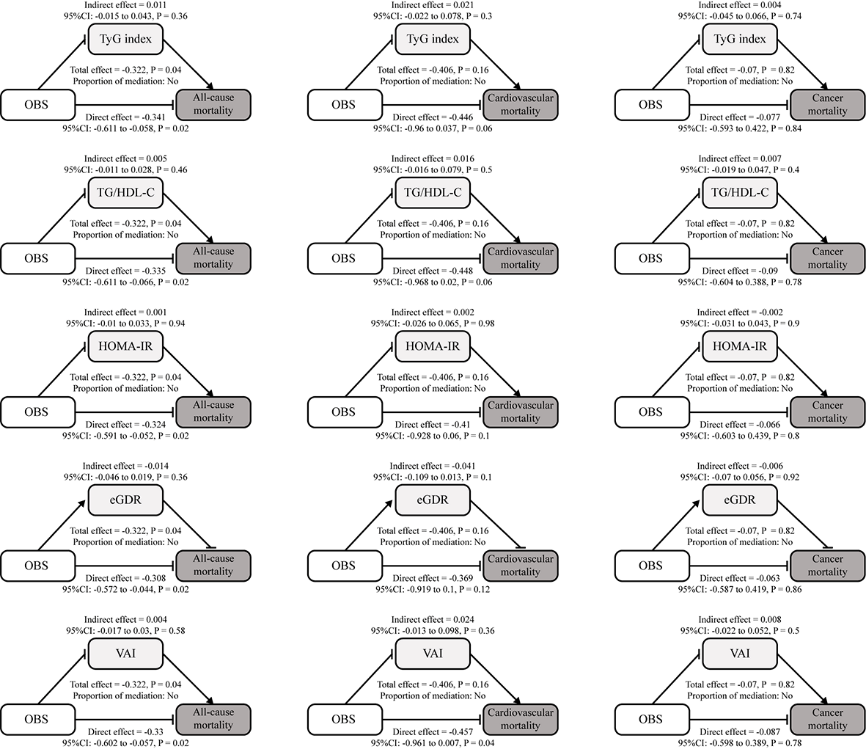


Figure S9: Sensitivity analysis of the mediation by insulin resistance indices of the associations of OBS with mortality in participants at least 65 years old.

Sensitivity analysis of the mediation by insulin resistance indices in the associations between OBS and mortality outcomes (all-cause, cardiovascular, and cancer) in participants aged 65 years or older. No significant mediation effects were observed for any insulin resistance indices in this age group (P ≥ 0.05).
