# Supplementary material for: Oxidative balance score and mortality: mediating role of insulin resistance across age strata in the NHANES cohort
Source: Front Nutr. 2025 Jun 9;12:1604696. doi: 10.3389/fnut.2025.1604696 (PMC12184654; doi:10.3389/fnut.2025.1604696)
Supplement: Supplementary file 1 [file Data_Sheet_1.zip › Supplementary materials/Table S1-S10.docx]

| Table S1: Oxidative balance score assignment scheme. | | | | | | | | | | | | | | |
| --- | --- | --- | --- | --- | --- | --- | --- | --- | --- | --- | --- | --- | --- | --- |
| OBS components | | Property | | Male | | | | | Female | | | | | |
|  |  |  |  | 0 | | 1 | 2 | | 0 | | 1 | | 2 | |
| Dietary OBS components | |  | |  | |  |  | |  | |  | |  | |
| Dietary fiber (g/d) | | A | | <12.56 | | 12.56-19.70 | ≥19.70 | | <10.10 | | 10.10-16.31 | | ≥16.31 | |
| Carotene (RE/d) | | A | | <98.83 | | 98.83-306.25 | ≥306.25 | | <98.08 | | 98.08-383.50 | | ≥383.50 | |
| Riboflavin (mg/d) | | A | | <1.79 | | 1.79-2.69 | ≥2.69 | | <1.34 | | 1.34-2.02 | | ≥2.02 | |
| Niacin (mg/d) | | A | | <20.65 | | 20.65-29.75 | ≥29.75 | | <14.52 | | 14.52-21.86 | | ≥21.86 | |
| Vitamin B6 (mg/d) | | A | | <1.59 | | 1.59-2.40 | ≥2.40 | | <1.13 | | 1.13-1.77 | | ≥1.77 | |
| Total folate (mcg/d) | | A | | <316.00 | | 316.00-492.00 | ≥492.00 | | <251.00 | | 251.00-388.96 | | ≥388.96 | |
| Vitamin B12 (mcg/d) | | A | | <3.36 | | 3.36-6.20 | ≥6.20 | | <2.22 | | 2.22-4.22 | | ≥4.22 | |
| Vitamin C (mg/d) | | A | | <42.44 | | 42.44-113.21 | ≥113.21 | | <38.01 | | 38.01-98.49 | | ≥98.49 | |
| Vitamin E (ATE) (mg/d) | | A | | <5.82 | | 5.82-9.42 | ≥9.42 | | <4.53 | | 4.53-7.52 | | ≥7.52 | |
| Calcium (mg/d) | | A | | <646.00 | | 646.00-1072.00 | ≥1072.00 | | <499.24 | | 499.24-849.00 | | ≥849.00 | |
| Magnesium (mg/d) | | A | | <257.00 | | 257.00-361.28 | ≥361.28 | | <187.00 | | 187.00-283.43 | | ≥283.43 | |
| Zinc (mg/d) | | A | | <9.75 | | 9.75-15.10 | ≥15.10 | | <6.73 | | 6.73-10.75 | | ≥10.75 | |
| Copper (mg/d) | | A | | <1.12 | | 1.12-1.57 | ≥1.57 | | <0.85 | | 0.85-1.28 | | ≥1.28 | |
| Selenium (mcg/d) | | A | | <94.94 | | 94.94-141.80 | ≥141.80 | | <67.79 | | 67.79-99.50 | | ≥99.50 | |
| Total fat (g/d) | | P | | ≥69.83 | | 69.83-107.43 | <107.43 | | ≥50.98 | | 50.98-75.79 | | <75.79 | |
| Iron (mg/d) | | P | | ≥12.88 | | 12.88-19.17 | <19.17 | | ≥9.65 | | 9.65-14.32 | | <14.32 | |
| Lifestyle OBS components | |  | |  | |  |  | |  | |  | |  | |
| Physical activity (MET-minute/week) | | A | | <417.86 | | 417.86-1135.71 | ≥1135.71 | | <270.00 | | 270.00-845.71 | | ≥845.71 | |
| Alcohol (g/d) | | P | | ≥30 | | 0-30 | None | | ≥15 | | 0-15 | | None | |
| Body mass index (kg/m2) | | P | | ≥25.54 | | 25.54-29.17 | <29.17 | | ≥23.74 | | 23.74-28.64 | | <28.64 | |
| Cotinine (ng/mL) | | P | | ≥0.038 | | 0.038-1.13 | <1.13 | | ≥0.035 | | 0.035-0.172 | | <0.172 | |
| Abbreviations: OBS, oxidative balance score; A, antioxidant; P, prooxidant; RE, retinol equivalent; ATE, alpha-tocopherol equivalent; MET, metabolic equivalent. | | | | | | | | | | | | | | |
| Table S2: Subgroup analysis of associations between OBS and all-cause mortality. | | | | | | | | | | | | | |  |
|  | Model 1 | | | | Model 2 | | | | | Model 3 | | | |  |
|  | HR (95% CI) | | P-Value | | HR (95% CI) | | | P-Value | | HR (95% CI) | | P-Value | |  |
| **All-cause mortality** |  | |  | |  | | |  | |  | |  | |  |
| **Sex** |  | |  | |  | | |  | |  | |  | |  |
| Male | 0.941 (0.929 to 0.955) | | < 0.001 | | 0.952 (0.938 to 0.966) | | | < 0.001 | | 0.973 (0.959 to 0.988) | | < 0.001 | |  |
| Female | 0.948 (0.931 to 0.965) | | < 0.001 | | 0.953 (0.937 to 0.969) | | | < 0.001 | | 0.973 (0.954 to 0.993) | | 0.009 | |  |
| **Age** |  | |  | |  | | |  | |  | |  | |  |
| <65 | 0.939 (0.917 to 0.961) | | < 0.001 | | 0.941 (0.920 to 0.963) | | | < 0.001 | | 0.966 (0.943 to 0.99) | | 0.005 | |  |
| ≥ 65 | 0.960 (0.951 to 0.971) | | < 0.001 | | 0.957 (0.947 to 0.968) | | | < 0.001 | | 0.977 (0.965 to 0.99) | | < 0.001 | |  |
| **Race** |  | |  | |  | | |  | |  | |  | |  |
| Non-Hispanic White | 0.940 (0.928 to 0.952) | | < 0.001 | | 0.945 (0.934 to 0.957) | | | < 0.001 | | 0.968 (0.955 to 0.982) | | < 0.001 | |  |
| Non-Hispanic Black | 0.969 (0.944 to 0.995) | | 0.019 | | 0.981 (0.956 to 1.007) | | | 0.147 | | 0.991 (0.964 to 1.02) | | 0.543 | |  |
| Other Race | 0.952 (0.931 to 0.972) | | < 0.001 | | 0.982 (0.959 to 1.005) | | | 0.120 | | 0.993 (0.970 to 1.016) | | 0.540 | |  |
| **Educational level** |  | |  | |  | | |  | |  | |  | |  |
| High school or below | 0.964 (0.951 to 0.977) | | < 0.001 | | 0.97 (0.957 to 0.983) | | | < 0.001 | | 0.979 (0.966 to 0.992) | | 0.002 | |  |
| Above high school | 0.942 (0.922 to 0.963) | | < 0.001 | | 0.947 (0.928 to 0.966) | | | < 0.001 | | 0.966 (0.945 to 0.986) | | 0.001 | |  |
| **Marital status** |  | |  | |  | | |  | |  | |  | |  |
| Never married | 0.945 (0.911 to 0.98) | | 0.002 | | 0.954 (0.918 to 0.991) | | | 0.016 | | 0.969 (0.932 to 1.007) | | 0.110 | |  |
| Married | 0.939 (0.924 to 0.955) | | < 0.001 | | 0.948 (0.932 to 0.963) | | | < 0.001 | | 0.967 (0.951 to 0.985) | | < 0.001 | |  |
| Other | 0.967 (0.950 to 0.984) | | < 0.001 | | 0.969 (0.952 to 0.985) | | | < 0.001 | | 0.985 (0.968 to 1.003) | | 0.106 | |  |
| **PIR** |  | |  | |  | | |  | |  | |  | |  |
| <1 | 0.952 (0.929 to 0.976) | | < 0.001 | | 0.968 (0.946 to 0.990) | | | 0.005 | | 0.978 (0.954 to 1.003) | | 0.088 | |  |
| 1 to <3.5 | 0.952 (0.938 to 0.966) | | < 0.001 | | 0.962 (0.95 to 0.975) | | | < 0.001 | | 0.974 (0.96 to 0.988) | | < 0.001 | |  |
| ≥3.5 | 0.955 (0.933 to 0.979) | | < 0.001 | | 0.958 (0.933 to 0.984) | | | 0.002 | | 0.969 (0.942 to 0.997) | | 0.028 | |  |
| **Household size** |  | |  | |  | | |  | |  | |  | |  |
| 1 to 2 | 0.942 (0.930 to 0.954) | | < 0.001 | | 0.949 (0.937 to 0.961) | | | < 0.001 | | 0.975 (0.961 to 0.988) | | < 0.001 | |  |
| ≥3 | 0.942 (0.922 to 0.962) | | < 0.001 | | 0.959 (0.938 to 0.98) | | | < 0.001 | | 0.972 (0.949 to 0.995) | | 0.016 | |  |
| **Sedentary** |  | |  | |  | | |  | |  | |  | |  |
| < 5 | 0.957 (0.938 to 0.975) | | < 0.001 | | 0.960 (0.942 to 0.978) | | | < 0.001 | | 0.983 (0.963 to 1.004) | | 0.107 | |  |
| 5 to <8 | 0.954 (0.937 to 0.971) | | < 0.001 | | 0.966 (0.948 to 0.983) | | | < 0.001 | | 0.984 (0.965 to 1.003) | | 0.099 | |  |
| ≥ 8 | 0.932 (0.915 to 0.949) | | < 0.001 | | 0.940 (0.922 to 0.958) | | | < 0.001 | | 0.960 (0.940 to 0.979) | | < 0.001 | |  |
| **Sleep duration** |  | |  | |  | | |  | |  | |  | |  |
| < 7 | 0.948 (0.935 to 0.962) | | < 0.001 | | 0.956 (0.941 to 0.971) | | | < 0.001 | | 0.969 (0.954 to 0.985) | | < 0.001 | |  |
| 7 to <8 | 0.939 (0.923 to 0.955) | | < 0.001 | | 0.947 (0.93 to 0.963) | | | < 0.001 | | 0.969 (0.951 to 0.988) | | 0.002 | |  |
| ≥ 8 | 0.969 (0.944 to 0.995) | | 0.021 | | 0.982 (0.957 to 1.009) | | | 0.196 | | 0.995 (0.968 to 1.022) | | 0.696 | |  |
| **Depression** |  | |  | |  | | |  | |  | |  | |  |
| <10 | 0.943 (0.932 to 0.954) | | < 0.001 | | 0.952 (0.941 to 0.963) | | | < 0.001 | | 0.973 (0.961 to 0.986) | | < 0.001 | |  |
| ≥ 10 | 0.973 (0.940 to 1.007) | | 0.124 | | 0.977 (0.942 to 1.013) | | | 0.204 | | 0.977 (0.943 to 1.013) | | 0.208 | |  |
| **Smoking** |  | |  | |  | | |  | |  | |  | |  |
| Never | 0.945 (0.927 to 0.962) | | < 0.001 | | 0.959 (0.941 to 0.978) | | | < 0.001 | | 0.976 (0.957 to 0.995) | | 0.015 | |  |
| Former or current | 0.955 (0.942 to 0.969) | | < 0.001 | | 0.955 (0.942 to 0.969) | | | < 0.001 | | 0.973 (0.958 to 0.988) | | 0.001 | |  |
| **Drinking** |  | |  | |  | | |  | |  | |  | |  |
| Never | 0.946 (0.923 to 0.969) | | < 0.001 | | 0.957 (0.936 to 0.978) | | | < 0.001 | | 0.967 (0.942 to 0.993) | | 0.012 | |  |
| Former or current | 0.945 (0.933 to 0.958) | | < 0.001 | | 0.952 (0.94 to 0.964) | | | < 0.001 | | 0.975 (0.962 to 0.988) | | < 0.001 | |  |

Analyses were conducted using weighted Cox models.

Abbreviations: PIR, poverty- income ratio; HR, hazard ratio; CI, confidence interval.

Model 1: not adjusted for covariates.

Model 2: adjusted for sex, age, and race.

Model 3: adjusted for sex, age, race, education, marital status, PIR, household size, sedentary duration, sleep duration, depression, smoking, and drinking.

| Table S3: Subgroup analysis of associations between OBS and cardiovascular mortality. | | | | | | |
| --- | --- | --- | --- | --- | --- | --- |
|  | Model 1 | | Model 2 | | Model 3 | |
|  | HR (95% CI) | P-Value | HR (95% CI) | P-Value | HR (95% CI) | P-Value |
| **Cardiovascular mortality** |  |  |  |  |  |  |
| **Sex** |  |  |  |  |  |  |
| Male | 0.939 (0.913 to 0.966) | < 0.001 | 0.948 (0.920 to 0.977) | < 0.001 | 0.966 (0.936 to 0.996) | 0.028 |
| Female | 0.948 (0.924 to 0.973) | < 0.001 | 0.959 (0.935 to 0.984) | 0.001 | 0.982 (0.951 to 1.013) | 0.248 |
| **Age** |  |  |  |  |  |  |
| <65 | 0.937 (0.893 to 0.984) | 0.009 | 0.939 (0.893 to 0.988) | 0.016 | 0.963 (0.915 to 1.014) | 0.154 |
| ≥ 65 | 0.959 (0.943 to 0.975) | < 0.001 | 0.956 (0.939 to 0.973) | < 0.001 | 0.977 (0.958 to 0.997) | 0.021 |
| **Race** |  |  |  |  |  |  |
| Non-Hispanic White | 0.937 (0.917 to 0.957) | < 0.001 | 0.943 (0.921 to 0.965) | < 0.001 | 0.965 (0.942 to 0.989) | 0.004 |
| Non-Hispanic Black | 0.963 (0.918 to 1.010) | 0.120 | 0.979 (0.936 to 1.024) | 0.345 | 0.993 (0.944 to 1.046) | 0.797 |
| Other Race | 0.986 (0.935 to 1.039) | 0.592 | 1.020 (0.963 to 1.08) | 0.505 | 1.027 (0.980 to 1.077) | 0.270 |
| **Educational level** |  |  |  |  |  |  |
| High school or below | 0.974 (0.948 to 1.000) | 0.049 | 0.982 (0.956 to 1.008) | 0.179 | 0.991 (0.966 to 1.017) | 0.509 |
| Above high school | 0.928 (0.899 to 0.957) | < 0.001 | 0.931 (0.900 to 0.963) | < 0.001 | 0.947 (0.917 to 0.977) | 0.001 |
| **Marital status** |  |  |  |  |  |  |
| Never married | 0.902 (0.853 to 0.955) | < 0.001 | 0.919 (0.863 to 0.977) | 0.007 | 0.924 (0.859 to 0.995) | 0.036 |
| Married | 0.926 (0.901 to 0.951) | < 0.001 | 0.934 (0.907 to 0.962) | < 0.001 | 0.951 (0.922 to 0.982) | 0.002 |
| Other | 0.993 (0.966 to 1.021) | 0.635 | 0.997 (0.970 to 1.026) | 0.846 | 1.014 (0.984 to 1.045) | 0.370 |
| **PIR** |  |  |  |  |  |  |
| <1 | 0.949 (0.913 to 0.986) | 0.008 | 0.968 (0.932 to 1.006) | 0.101 | 0.977 (0.94 to 1.015) | 0.233 |
| 1 to <3.5 | 0.955 (0.930 to 0.981) | 0.001 | 0.969 (0.945 to 0.992) | 0.010 | 0.987 (0.960 to 1.015) | 0.349 |
| ≥3.5 | 0.943 (0.900 to 0.988) | 0.014 | 0.943 (0.895 to 0.994) | 0.030 | 0.943 (0.897 to 0.992) | 0.023 |
| **Household size** |  |  |  |  |  |  |
| 1 to 2 | 0.940 (0.921 to 0.959) | < 0.001 | 0.948 (0.928 to 0.969) | < 0.001 | 0.971 (0.950 to 0.993) | 0.009 |
| ≥3 | 0.944 (0.909 to 0.979) | 0.002 | 0.970 (0.928 to 1.013) | 0.171 | 0.991 (0.950 to 1.034) | 0.670 |
| **Sedentary** |  |  |  |  |  |  |
| < 5 | 0.976 (0.937 to 1.016) | 0.235 | 0.985 (0.946 to 1.025) | 0.454 | 1.008 (0.968 to 1.049) | 0.715 |
| 5 to <8 | 0.945 (0.909 to 0.982) | 0.004 | 0.960 (0.919 to 1.003) | 0.070 | 0.974 (0.926 to 1.024) | 0.300 |
| ≥ 8 | 0.925 (0.906 to 0.945) | < 0.001 | 0.931 (0.910 to 0.953) | < 0.001 | 0.955 (0.929 to 0.981) | 0.001 |
| **Sleep duration** |  |  |  |  |  |  |
| < 7 | 0.952 (0.928 to 0.976) | < 0.001 | 0.960 (0.931 to 0.991) | 0.011 | 0.976 (0.943 to 1.011) | 0.175 |
| 7 to <8 | 0.939 (0.910 to 0.968) | < 0.001 | 0.948 (0.916 to 0.980) | 0.002 | 0.963 (0.933 to 0.994) | 0.020 |
| ≥ 8 | 0.964 (0.912 to 1.018) | 0.189 | 0.978 (0.928 to 1.029) | 0.388 | 1.001 (0.951 to 1.053) | 0.980 |
| **Depression** |  |  |  |  |  |  |
| <10 | 0.937 (0.920 to 0.955) | < 0.001 | 0.948 (0.929 to 0.968) | < 0.001 | 0.968 (0.947 to 0.988) | 0.002 |
| ≥ 10 | 1.013 (0.953 to 1.076) | 0.677 | 1.024 (0.953 to 1.101) | 0.515 | 1.032 (0.972 to 1.095) | 0.307 |
| **Smoking** |  |  |  |  |  |  |
| Never | 0.966 (0.939 to 0.994) | 0.017 | 0.986 (0.959 to 1.013) | 0.308 | 1.002 (0.974 to 1.032) | 0.878 |
| Former or current | 0.930 (0.907 to 0.953) | < 0.001 | 0.927 (0.902 to 0.953) | < 0.001 | 0.949 (0.922 to 0.977) | < 0.001 |
| **Drinking** |  |  |  |  |  |  |
| Never | 0.973 (0.929 to 1.018) | 0.232 | 0.987 (0.945 to 1.030) | 0.543 | 1.004 (0.952 to 1.060) | 0.874 |
| Former or current | 0.939 (0.920 to 0.959) | < 0.001 | 0.947 (0.926 to 0.968) | < 0.001 | 0.969 (0.947 to 0.991) | 0.006 |

Analyses were conducted using weighted Cox models.

Abbreviations: PIR, poverty- income ratio; HR, hazard ratio; CI, confidence interval.

Model 1: not adjusted for covariates.

Model 2: adjusted for sex, age, and race.

Model 3: adjusted for sex, age, race, education, marital status, PIR, household size, sedentary duration, sleep duration, depression, smoking, and drinking.

| Table S4: Subgroup analysis of associations between OBS and cancer mortality. | | | | | | |
| --- | --- | --- | --- | --- | --- | --- |
|  | Model 1 | | Model 2 | | Model 3 | |
|  | HR (95% CI) | P-Value | HR (95% CI) | P-Value | HR (95% CI) | P-Value |
| **Cancer mortality** |  |  |  |  |  |  |
| **Sex** |  |  |  |  |  |  |
| Male | 0.960 (0.933 to 0.988) | 0.006 | 0.973 (0.944 to 1.002) | 0.070 | 0.987 (0.956 to 1.018) | 0.404 |
| Female | 0.959 (0.932 to 0.988) | 0.006 | 0.963 (0.935 to 0.993) | 0.015 | 0.985 (0.958 to 1.013) | 0.284 |
| **Age** |  |  |  |  |  |  |
| <65 | 0.957 (0.919 to 0.997) | 0.036 | 0.959 (0.920 to 1.000) | 0.052 | 0.978 (0.938 to 1.019) | 0.288 |
| ≥ 65 | 0.975 (0.955 to 0.996) | 0.019 | 0.972 (0.951 to 0.994) | 0.014 | 0.99 (0.969 to 1.013) | 0.394 |
| **Race** |  |  |  |  |  |  |
| Non-Hispanic White | 0.959 (0.94 to 0.979) | < 0.001 | 0.965 (0.945 to 0.986) | 0.001 | 0.986 (0.964 to 1.008) | 0.209 |
| Non-Hispanic Black | 0.978 (0.939 to 1.019) | 0.286 | 0.993 (0.953 to 1.035) | 0.743 | 0.991 (0.950 to 1.035) | 0.694 |
| Other Race | 0.930 (0.886 to 0.975) | 0.003 | 0.954 (0.907 to 1.005) | 0.075 | 0.962 (0.911 to 1.015) | 0.156 |
| **Educational level** |  |  |  |  |  |  |
| High school or below | 0.966 (0.942 to 0.991) | 0.008 | 0.972 (0.946 to 0.998) | 0.036 | 0.981 (0.958 to 1.006) | 0.130 |
| Above high school | 0.971 (0.937 to 1.006) | 0.105 | 0.977 (0.943 to 1.013) | 0.211 | 0.99 (0.955 to 1.027) | 0.607 |
| **Marital status** |  |  |  |  |  |  |
| Never married | 0.966 (0.908 to 1.028) | 0.280 | 0.967 (0.905 to 1.034) | 0.330 | 0.966 (0.899 to 1.038) | 0.348 |
| Married | 0.960 (0.935 to 0.986) | 0.003 | 0.969 (0.941 to 0.996) | 0.027 | 0.986 (0.960 to 1.013) | 0.312 |
| Other | 0.971 (0.939 to 1.004) | 0.083 | 0.974 (0.941 to 1.008) | 0.130 | 0.988 (0.954 to 1.023) | 0.494 |
| **PIR** |  |  |  |  |  |  |
| <1 | 0.938 (0.894 to 0.985) | 0.009 | 0.952 (0.909 to 0.997) | 0.035 | 0.967 (0.923 to 1.013) | 0.152 |
| 1 to <3.5 | 0.959 (0.936 to 0.983) | 0.001 | 0.969 (0.945 to 0.992) | 0.010 | 0.975 (0.952 to 0.999) | 0.040 |
| ≥3.5 | 0.999 (0.956 to 1.044) | 0.950 | 1.008 (0.961 to 1.057) | 0.743 | 1.023 (0.969 to 1.081) | 0.410 |
| **Household size** |  |  |  |  |  |  |
| 1 to 2 | 0.961 (0.941 to 0.981) | < 0.001 | 0.970 (0.948 to 0.992) | 0.009 | 0.994 (0.972 to 1.016) | 0.582 |
| ≥3 | 0.947 (0.905 to 0.991) | 0.019 | 0.960 (0.916 to 1.005) | 0.080 | 0.969 (0.925 to 1.014) | 0.176 |
| **Sedentary** |  |  |  |  |  |  |
| < 5 | 0.960 (0.933 to 0.988) | 0.005 | 0.960 (0.930 to 0.991) | 0.013 | 0.972 (0.936 to 1.008) | 0.130 |
| 5 to <8 | 0.958 (0.924 to 0.994) | 0.021 | 0.970 (0.935 to 1.006) | 0.101 | 0.991 (0.955 to 1.028) | 0.629 |
| ≥ 8 | 0.962 (0.925 to 1.001) | 0.058 | 0.974 (0.936 to 1.013) | 0.186 | 0.993 (0.955 to 1.033) | 0.743 |
| **Sleep duration** |  |  |  |  |  |  |
| < 7 | 0.953 (0.922 to 0.985) | 0.004 | 0.962 (0.929 to 0.996) | 0.031 | 0.97 (0.938 to 1.004) | 0.081 |
| 7 to <8 | 0.971 (0.942 to 1.001) | 0.054 | 0.978 (0.949 to 1.008) | 0.149 | 1.002 (0.972 to 1.034) | 0.876 |
| ≥ 8 | 0.948 (0.905 to 0.994) | 0.028 | 0.960 (0.913 to 1.010) | 0.115 | 0.951 (0.898 to 1.007) | 0.084 |
| **Depression** |  |  |  |  |  |  |
| <10 | 0.959 (0.94 to 0.978) | < 0.001 | 0.967 (0.946 to 0.988) | 0.002 | 0.987 (0.965 to 1.008) | 0.224 |
| ≥ 10 | 0.970 (0.899 to 1.046) | 0.426 | 0.970 (0.891 to 1.056) | 0.481 | 0.959 (0.845 to 1.089) | 0.519 |
| **Smoking** |  |  |  |  |  |  |
| Never | 0.957 (0.930 to 0.986) | 0.004 | 0.97 (0.938 to 1.003) | 0.078 | 0.983 (0.95 to 1.017) | 0.327 |
| Former or current | 0.974 (0.951 to 0.998) | 0.032 | 0.975 (0.952 to 0.999) | 0.040 | 0.990 (0.966 to 1.014) | 0.413 |
| **Drinking** |  |  |  |  |  |  |
| Never | 0.966 (0.924 to 1.010) | 0.132 | 0.976 (0.923 to 1.031) | 0.384 | 0.982 (0.932 to 1.034) | 0.495 |
| Former or current | 0.960 (0.941 to 0.979) | < 0.001 | 0.967 (0.947 to 0.988) | 0.002 | 0.987 (0.965 to 1.009) | 0.245 |

Analyses were conducted using weighted Cox models.

Abbreviations: PIR, poverty- income ratio; HR, hazard ratio; CI, confidence interval.

Model 1: not adjusted for covariates.

Model 2: adjusted for sex, age, and race.

Model 3: adjusted for sex, age, race, education, marital status, PIR, household size, sedentary duration, sleep duration, depression, smoking, and drinking.

Table S5: The associations between OBS and insulin resistance indices for all included participants

|  | Model 1 | | Model 2 | | Model 3 | |
| --- | --- | --- | --- | --- | --- | --- |
|  | β (95% CI) | P-Value | β (95% CI) | P-Value | β (95% CI) | P-Value |
| TyG index |  |  |  |  |  |  |
| OBS tertile 1 | Reference |  | Reference |  | Reference |  |
| OBS tertile 2 | -0.049 (-0.090 to -0.009) | 0.018 | -0.067 (-0.108 to -0.026) | 0.002 | -0.040 (-0.080 to 0.000) | 0.048 |
| OBS tertile 3 | -0.148 (-0.190 to -0.106) | < 0.001 | -0.170 (-0.214 to -0.125) | < 0.001 | -0.121 (-0.163 to -0.079) | < 0.001 |
| TG/HDL-C |  |  |  |  |  |  |
| OBS tertile 1 | Reference |  | Reference |  | Reference |  |
| OBS tertile 2 | -0.084 (-0.268 to 0.099) | 0.365 | -0.171 (-0.358 to 0.015) | 0.071 | -0.059 (-0.247 to 0.129) | 0.534 |
| OBS tertile 3 | -0.374 (-0.546 to -0.201) | < 0.001 | -0.499 (-0.68 to -0.318) | < 0.001 | -0.304 (-0.492 to -0.116) | 0.002 |
| HOMA-IR |  |  |  |  |  |  |
| OBS tertile 1 | Reference |  | Reference |  | Reference |  |
| OBS tertile 2 | -0.468 (-0.812 to -0.125) | 0.008 | -0.461 (-0.802 to -0.120) | 0.009 | -0.307 (-0.646 to 0.033) | 0.076 |
| OBS tertile 3 | -1.164 (-1.449 to -0.879) | < 0.001 | -1.119 (-1.408 to -0.829) | < 0.001 | -0.836 (-1.130 to -0.541) | < 0.001 |
| eGDR |  |  |  |  |  |  |
| OBS tertile 1 | Reference |  | Reference |  | Reference |  |
| OBS tertile 2 | 0.233 (0.052 to 0.414) | 0.012 | 0.164 (-0.008 to 0.335) | 0.061 | 0.051 (-0.116 to 0.218) | 0.543 |
| OBS tertile 3 | 0.950 (0.791 to 1.109) | < 0.001 | 0.764 (0.591 to 0.936) | < 0.001 | 0.562 (0.398 to 0.726) | < 0.001 |
| VAI |  |  |  |  |  |  |
| OBS tertile 1 | Reference |  | Reference |  | Reference |  |
| OBS tertile 2 | -0.045 (-0.108 to 0.018) | 0.163 | -0.065 (-0.130 to -0.001) | 0.048 | -0.023 (-0.088 to 0.042) | 0.485 |
| OBS tertile 3 | -0.149 (-0.209 to -0.089) | < 0.001 | -0.185 (-0.248 to -0.123) | < 0.001 | -0.113 (-0.176 to -0.049) | 0.001 |

Analyses were conducted using weighted multiple linear regression models.

Abbreviations: TyG index, triglyceride-glucose index; TG/HDL-C, triglyceride/high-density lipoprotein cholesterol ratio; HOMA-IR, homeostatic model assessment of insulin resistance; eGDR, estimated glucose disposal rate; VAI, visceral adiposity index; CI, confidence interval.

Model 1: not adjusted for covariates.

Model 2: adjusted for sex, age, and race.

Model 3: adjusted for sex, age, race, education, marital status, PIR, household size, sedentary duration, sleep duration, depression, smoking, and drinking.

Table S6: The associations between OBS and insulin resistance indices for participants younger than 65 years.

|  | Model 1 | | Model 2 | | Model 3 | |
| --- | --- | --- | --- | --- | --- | --- |
|  | β (95% CI) | P-Value | β (95% CI) | P-Value | β (95% CI) | P-Value |
| TyG index |  |  |  |  |  |  |
| OBS tertile 1 | Reference |  | Reference |  | Reference |  |
| OBS tertile 2 | -0.041 (-0.089 to 0.008) | 0.099 | -0.067 (-0.116 to -0.018) | 0.008 | -0.041 (-0.09 to 0.007) | 0.095 |
| OBS tertile 3 | -0.129 (-0.183 to -0.076) | < 0.001 | -0.168 (-0.223 to -0.113) | < 0.001 | -0.122 (-0.175 to -0.068) | < 0.001 |
| TG/HDL-C |  |  |  |  |  |  |
| OBS tertile 1 | Reference |  | Reference |  | Reference |  |
| OBS tertile 2 | -0.054 (-0.268 to 0.159) | 0.615 | -0.154 (-0.371 to 0.064) | 0.164 | -0.041 (-0.264 to 0.182) | 0.712 |
| OBS tertile 3 | -0.390 (-0.605 to -0.175) | 0.001 | -0.525 (-0.749 to -0.301) | < 0.001 | -0.325 (-0.557 to -0.093) | 0.007 |
| HOMA-IR |  |  |  |  |  |  |
| OBS tertile 1 | Reference |  | Reference |  | Reference |  |
| OBS tertile 2 | -0.455 (-0.899 to -0.011) | 0.045 | -0.474 (-0.922 to -0.025) | 0.039 | -0.337 (-0.788 to 0.113) | 0.140 |
| OBS tertile 3 | -1.160 (-1.518 to -0.803) | < 0.001 | -1.166 (-1.530 to -0.802) | < 0.001 | -0.914 (-1.289 to -0.538) | < 0.001 |
| eGDR |  |  |  |  |  |  |
| OBS tertile 1 | Reference |  | Reference |  | Reference |  |
| OBS tertile 2 | 0.161 (-0.04 to 0.362) | 0.115 | 0.141 (-0.059 to 0.34) | 0.166 | 0.035 (-0.156 to 0.226) | 0.716 |
| OBS tertile 3 | 0.778 (0.584 to 0.973) | < 0.001 | 0.719 (0.519 to 0.919) | < 0.001 | 0.533 (0.343 to 0.723) | < 0.001 |
| VAI |  |  |  |  |  |  |
| OBS tertile 1 | Reference |  | Reference |  | Reference |  |
| OBS tertile 2 | -0.038 (-0.111 to 0.036) | 0.310 | -0.059 (-0.134 to 0.017) | 0.125 | -0.017 (-0.094 to 0.061) | 0.671 |
| OBS tertile 3 | -0.153 (-0.228 to -0.079) | < 0.001 | -0.191 (-0.268 to -0.113) | < 0.001 | -0.117 (-0.195 to -0.038) | 0.004 |

Analyses were conducted using weighted multiple linear regression models.

Abbreviations: TyG index, triglyceride-glucose index; TG/HDL-C, triglyceride/high-density lipoprotein cholesterol ratio; HOMA-IR, homeostatic model assessment of insulin resistance; eGDR, estimated glucose disposal rate; VAI, visceral adiposity index; CI, confidence interval.

Model 1: not adjusted for covariates.

Model 2: adjusted for sex, age, and race.

Model 3: adjusted for sex, age, race, education, marital status, PIR, household size, sedentary duration, sleep duration, depression, smoking, and drinking.

Table S7: The associations between OBS and insulin resistance indices for participants at least 65 years old.

|  | Model 1 | | Model 2 | | Model 3 | |
| --- | --- | --- | --- | --- | --- | --- |
|  | β (95% CI) | P-Value | β (95% CI) | P-Value | β (95% CI) | P-Value |
| TyG index |  |  |  |  |  |  |
| OBS tertile 1 | Reference |  | Reference |  | Reference |  |
| OBS tertile 2 | -0.06 (-0.125 to 0.004) | 0.066 | -0.078 (-0.141 to -0.015) | 0.016 | -0.039 (-0.098 to 0.019) | 0.186 |
| OBS tertile 3 | -0.170 (-0.256 to -0.084) | < 0.001 | -0.189 (-0.276 to -0.103) | < 0.001 | -0.127 (-0.215 to -0.038) | 0.005 |
| TG/HDL-C |  |  |  |  |  |  |
| OBS tertile 1 | Reference |  | Reference |  | Reference |  |
| OBS tertile 2 | -0.229 (-0.497 to 0.038) | 0.092 | -0.277 (-0.542 to -0.012) | 0.041 | -0.179 (-0.431 to 0.072) | 0.160 |
| OBS tertile 3 | -0.387 (-0.677 to -0.098) | 0.009 | -0.426 (-0.720 to -0.131) | 0.005 | -0.270 (-0.581 to 0.041) | 0.088 |
| HOMA-IR |  |  |  |  |  |  |
| OBS tertile 1 | Reference |  | Reference |  | Reference |  |
| OBS tertile 2 | -0.467 (-1.342 to 0.407) | 0.291 | -0.435 (-1.296 to 0.426) | 0.319 | -0.259 (-1.11 to 0.593) | 0.547 |
| OBS tertile 3 | -0.994 (-1.777 to -0.211) | 0.013 | -0.924 (-1.707 to -0.141) | 0.021 | -0.542 (-1.285 to 0.201) | 0.151 |
| eGDR |  |  |  |  |  |  |
| OBS tertile 1 | Reference |  | Reference |  | Reference |  |
| OBS tertile 2 | 0.28 (0.024 to 0.536) | 0.032 | 0.225 (-0.025 to 0.475) | 0.077 | 0.059 (-0.196 to 0.314) | 0.644 |
| OBS tertile 3 | 1.016 (0.735 to 1.298) | < 0.001 | 0.929 (0.649 to 1.209) | < 0.001 | 0.639 (0.352 to 0.927) | < 0.001 |
| VAI |  |  |  |  |  |  |
| OBS tertile 1 | Reference |  | Reference |  | Reference |  |
| OBS tertile 2 | -0.076 (-0.178 to 0.026) | 0.142 | -0.101 (-0.202 to 0) | 0.051 | -0.062 (-0.157 to 0.033) | 0.195 |
| OBS tertile 3 | -0.137 (-0.241 to -0.032) | 0.011 | -0.172 (-0.277 to -0.066) | 0.002 | -0.109 (-0.221 to 0.003) | 0.057 |

Analyses were conducted using weighted multiple linear regression models.

Abbreviations: TyG index, triglyceride-glucose index; TG/HDL-C, triglyceride/high-density lipoprotein cholesterol ratio; HOMA-IR, homeostatic model assessment of insulin resistance; eGDR, estimated glucose disposal rate; VAI, visceral adiposity index; CI, confidence interval.

Model 1: not adjusted for covariates.

Model 2: adjusted for sex, age, and race.

Model 3: adjusted for sex, age, race, education, marital status, PIR, household size, sedentary duration, sleep duration, depression, smoking, and drinking.

Table S8: The associations of insulin resistance indices with in all included participants.

|  | Model 1 | | Model 2 | | Model 3 | |
| --- | --- | --- | --- | --- | --- | --- |
|  | HR (95% CI) | P-Value | HR (95% CI) | P-Value | HR (95% CI) | P-Value |
| All-cause mortality |  |  |  |  |  |  |
| TyG index | 1.537 (1.379 to 1.714) | < 0.001 | 1.376 (1.199 to 1.579) | < 0.001 | 1.148 (0.994 to 1.324) | 0.060 |
| TG/HDL-C | 1.008 (0.998 to 1.018) | 0.112 | 1.018 (1.008 to 1.029) | < 0.001 | 1.001 (0.983 to 1.019) | 0.934 |
| HOMA-IR | 1.014 (1.008 to 1.019) | < 0.001 | 1.014 (1.009 to 1.019) | < 0.001 | 1.012 (1.005 to 1.019) | 0.001 |
| eGDR | 0.799 (0.780 to 0.817) | < 0.001 | 0.872 (0.843 to 0.902) | < 0.001 | 0.909 (0.878 to 0.940) | < 0.001 |
| VAI | 1.029 (1.005 to 1.055) | 0.020 | 1.051 (1.024 to 1.08) | < 0.001 | 1.015 (0.974 to 1.057) | 0.490 |
| Cardiovascular mortality |  |  |  |  |  |  |
| TyG index | 1.586 (1.36 to 1.849) | < 0.001 | 1.394 (1.149 to 1.69) | 0.001 | 1.17 (0.958 to 1.43) | 0.124 |
| TG/HDL-C | 0.998 (0.975 to 1.021) | 0.846 | 1.007 (0.979 to 1.037) | 0.609 | 0.978 (0.93 to 1.028) | 0.375 |
| HOMA-IR | 1.015 (1.008 to 1.022) | < 0.001 | 1.016 (1.006 to 1.025) | 0.001 | 1.014 (1.001 to 1.028) | 0.031 |
| eGDR | 0.746 (0.716 to 0.777) | < 0.001 | 0.808 (0.755 to 0.864) | < 0.001 | 0.844 (0.788 to 0.905) | < 0.001 |
| VAI | 0.992 (0.935 to 1.052) | 0.779 | 1.026 (0.969 to 1.087) | 0.379 | 0.933 (0.815 to 1.069) | 0.318 |
| Cancer mortality |  |  |  |  |  |  |
| TyG index | 1.374 (1.168 to 1.616) | 0.000 | 1.193 (0.970 to 1.469) | 0.095 | 1.021 (0.81 to 1.287) | 0.859 |
| TG/HDL-C | 0.997 (0.976 to 1.019) | 0.795 | 1.008 (0.985 to 1.031) | 0.484 | 0.983 (0.940 to 1.028) | 0.456 |
| HOMA-IR | 1.009 (1.002 to 1.017) | 0.018 | 1.006 (0.994 to 1.019) | 0.312 | 1.002 (0.985 to 1.020) | 0.828 |
| eGDR | 0.846 (0.808 to 0.886) | 0.000 | 0.948 (0.895 to 1.005) | 0.073 | 0.985 (0.927 to 1.047) | 0.632 |
| VAI | 1.011 (0.969 to 1.054) | 0.616 | 1.034 (0.991 to 1.079) | 0.126 | 0.974 (0.861 to 1.102) | 0.677 |

Analyses were conducted using weighted multiple linear regression models.

Abbreviations: TyG index, triglyceride-glucose index; TG/HDL-C, triglyceride/high-density lipoprotein cholesterol ratio; HOMA-IR, homeostatic model assessment of insulin resistance; eGDR, estimated glucose disposal rate; VAI, visceral adiposity index; HR, hazard ratio; CI, confidence interval.

Model 1: not adjusted for covariates.

Model 2: adjusted for sex, age, and race.

Model 3: adjusted for sex, age, race, education, marital status, PIR, household size, sedentary duration, sleep duration, depression, smoking, and drinking.

| Table S9: The associations of insulin resistance indices with mortality in participants younger than 65 years. | | | | | | |
| --- | --- | --- | --- | --- | --- | --- |
|  | Model 1 | | Model 2 | | Model 3 | |
|  | HR (95% CI) | P-Value | HR (95% CI) | P-Value | HR (95% CI) | P-Value |
| All-cause mortality |  |  |  |  |  |  |
| TyG index | 1.706 (1.355 to 2.146) | < 0.001 | 1.746 (1.4 to 2.177) | < 0.001 | 1.536 (1.179 to 2.001) | 0.001 |
| TG/HDL-C | 1.014 (1.003 to 1.026) | 0.013 | 1.015 (1.003 to 1.027) | 0.013 | 1.005 (0.989 to 1.021) | 0.51 |
| HOMA-IR | 1.018 (1.011 to 1.025) | < 0.001 | 1.018 (1.012 to 1.025) | < 0.001 | 1.019 (1.011 to 1.027) | < 0.001 |
| eGDR | 0.792 (0.752 to 0.833) | < 0.001 | 0.804 (0.763 to 0.848) | < 0.001 | 0.827 (0.78 to 0.877) | < 0.001 |
| VAI | 1.035 (1.006 to 1.064) | 0.017 | 1.043 (1.014 to 1.072) | 0.003 | 1.009 (0.968 to 1.052) | 0.681 |
| Cardiovascular mortality |  |  |  |  |  |  |
| TyG index | 2.123 (1.520 to 2.965) | < 0.001 | 2.024 (1.456 to 2.814) | < 0.001 | 1.872 (1.199 to 2.922) | 0.006 |
| TG/HDL-C | 1.009 (0.990 to 1.028) | 0.362 | 1.002 (0.967 to 1.038) | 0.925 | 0.994 (0.956 to 1.033) | 0.746 |
| HOMA-IR | 1.021 (1.012 to 1.030) | < 0.001 | 1.020 (1.012 to 1.029) | < 0.001 | 1.022 (1.011 to 1.034) | < 0.001 |
| eGDR | 0.673 (0.595 to 0.763) | < 0.001 | 0.683 (0.601 to 0.777) | < 0.001 | 0.700 (0.602 to 0.813) | < 0.001 |
| VAI | 1.019 (0.962 to 1.078) | 0.525 | 1.035 (0.983 to 1.090) | 0.192 | 0.968 (0.824 to 1.137) | 0.694 |
| Cancer mortality |  |  |  |  |  |  |
| TyG index | 1.291 (0.953 to 1.749) | 0.099 | 1.338 (0.990 to 1.809) | 0.058 | 1.173 (0.827 to 1.665) | 0.371 |
| TG/HDL-C | 0.999 (0.969 to 1.031) | 0.966 | 1.000 (0.970 to 1.032) | 0.986 | 0.987 (0.944 to 1.032) | 0.564 |
| HOMA-IR | 1.004 (0.980 to 1.028) | 0.740 | 1.005 (0.980 to 1.030) | 0.709 | 1.000 (0.962 to 1.039) | 0.984 |
| eGDR | 0.910 (0.847 to 0.977) | 0.009 | 0.925 (0.861 to 0.993) | 0.031 | 0.955 (0.885 to 1.030) | 0.234 |
| VAI | 1.007 (0.938 to 1.082) | 0.838 | 1.017 (0.959 to 1.078) | 0.573 | 0.966 (0.838 to 1.113) | 0.631 |

Analyses were conducted using weighted Cox models.

Abbreviations: TyG index, triglyceride-glucose index; TG/HDL-C, triglyceride/high-density lipoprotein cholesterol ratio; HOMA-IR, homeostatic model assessment of insulin resistance; eGDR, estimated glucose disposal rate; VAI, visceral adiposity index; HR, hazard ratio; CI, confidence interval.

Model 1: not adjusted for covariates.

Model 2: adjusted for sex, age, and race.

Model 3: adjusted for sex, age, race, education, marital status, PIR, household size, sedentary duration, sleep duration, depression, smoking, and drinking.

Table S10: The associations of insulin resistance indices with mortality in participants at least 65 years old.

|  | Model 1 | | Model 2 | | Model 3 | |
| --- | --- | --- | --- | --- | --- | --- |
|  | HR (95% CI) | P-Value | HR (95% CI) | P-Value | HR (95% CI) | P-Value |
| All-cause mortality |  |  |  |  |  |  |
| TyG index | 1.144 (0.955 to 1.370) | 0.144 | 1.151 (0.961 to 1.379) | 0.128 | 0.959 (0.794 to 1.156) | 0.658 |
| TG/HDL-C | 1.043 (0.994 to 1.095) | 0.089 | 1.036 (0.984 to 1.090) | 0.183 | 0.999 (0.942 to 1.060) | 0.983 |
| HOMA-IR | 1.009 (0.997 to 1.021) | 0.156 | 1.008 (0.995 to 1.021) | 0.216 | 1.000 (0.983 to 1.018) | 0.978 |
| eGDR | 0.928 (0.890 to 0.967) | < 0.001 | 0.931 (0.891 to 0.973) | 0.002 | 0.964 (0.922 to 1.007) | 0.099 |
| VAI | 1.110 (0.953 to 1.294) | 0.181 | 1.132 (0.975 to 1.315) | 0.103 | 0.961 (0.796 to 1.161) | 0.680 |
| Cardiovascular mortality |  |  |  |  |  |  |
| TyG index | 1.021 (0.802 to 1.301) | 0.865 | 1.046 (0.83 to 1.318) | 0.703 | 0.884 (0.695 to 1.124) | 0.314 |
| TG/HDL-C | 1.006 (0.913 to 1.109) | 0.902 | 0.997 (0.906 to 1.097) | 0.950 | 0.973 (0.869 to 1.089) | 0.630 |
| HOMA-IR | 1.008 (0.986 to 1.030) | 0.473 | 1.006 (0.983 to 1.029) | 0.596 | 1.000 (0.968 to 1.033) | 0.997 |
| eGDR | 0.884 (0.823 to 0.951) | 0.001 | 0.894 (0.826 to 0.966) | 0.005 | 0.916 (0.850 to 0.987) | 0.021 |
| VAI | 0.912 (0.695 to 1.198) | 0.510 | 0.962 (0.751 to 1.231) | 0.756 | 0.777 (0.568 to 1.064) | 0.116 |
| Cancer mortality |  |  |  |  |  |  |
| TyG index | 1.098 (0.807 to 1.493) | 0.551 | 1.113 (0.822 to 1.507) | 0.489 | 0.974 (0.716 to 1.327) | 0.868 |
| TG/HDL-C | 1.035 (0.969 to 1.105) | 0.303 | 1.028 (0.962 to 1.097) | 0.417 | 1.002 (0.924 to 1.086) | 0.969 |
| HOMA-IR | 1.008 (0.995 to 1.022) | 0.237 | 1.007 (0.993 to 1.022) | 0.324 | 1.003 (0.984 to 1.024) | 0.741 |
| eGDR | 0.959 (0.892 to 1.031) | 0.260 | 0.967 (0.897 to 1.041) | 0.370 | 0.996 (0.921 to 1.077) | 0.925 |
| VAI | 1.079 (0.873 to 1.334) | 0.481 | 1.11 (0.910 to 1.352) | 0.304 | 0.978 (0.763 to 1.253) | 0.859 |

Analyses were conducted using weighted Cox models.

Abbreviations: TyG index, triglyceride-glucose index; TG/HDL-C, triglyceride/high-density lipoprotein cholesterol ratio; HOMA-IR, homeostatic model assessment of insulin resistance; eGDR, estimated glucose disposal rate; VAI, visceral adiposity index; HR, hazard ratio; CI, confidence interval.

Model 1: not adjusted for covariates.

Model 2: adjusted for sex, age, and race.

Model 3: adjusted for sex, age, race, education, marital status, PIR, household size, sedentary duration, sleep duration, depression, smoking, and drinking.
